# Unusual Failures Associated with Cracks That Nucleated from Corrosion Damage in Fatigue Tests on AA 7085-T7452 Specimens

**DOI:** 10.3390/ma19153301

**Published:** 2026-08-04

**Authors:** Daren Peng, Andrew S. M. Ang, Nam Phan, Michael R. Brindza, Ben Main, Rhys Jones

**Affiliations:** 1 ARC Industrial Transformation Training Centre on Surface Engineering for Advanced Materials, School of Engineering, Swinburne University of Technology, John Street, Hawthorn, VIC 3122, Australia; daren.peng@monash.edu (D.P.); aang@swin.edu.au (A.S.M.A.); 2 Department of Mechanical and Aerospace Engineering, Monash University, Clayton, VIC 3800, Australia; 3 Structures Division, Naval Air Systems Command, Patuxent River, MD 20670, USA; 4 Air Warfare & Weapons Department, Air Platforms Division, Office of Naval Research, Arlington, VA 22203-1995, USA; michael.r.brindza.civ@us.navy.mil; 5 Platforms Division, Defence Science and Technology Group, 506 Lorimer Street, Fishermans Bend, VIC 3207, Australia; ben.main1@defence.gov.au

**Keywords:** corrosion pits, ASTM B117-19, fatigue crack growth, 7085-T7452, intergranular cracking

## Abstract

The paper is the first to highlight what appears to be a unique and unusual failure mechanism that is associated with cracks that nucleated from corrosion pits in aluminium alloy (AA) 7085-T7452 specimens that were tested under a variable amplitude load spectrum. In this study, cracks initially nucleated at corrosion pits and first grew as would be expected, namely at ninety degrees to the surface and perpendicular to the applied load. However, after reaching a depth of approximately 2 mm, these various Mode I cracks transformed into what can be best described as interlayer cracks with their surfaces at an angle of approximately ninety degrees to the initial fatigue crack surface. Analysis of the failures revealed that the maximum value of the stress intensity factor at which this phenomenon occurred, which we have defined as *K_IL_*, was substantially less than the fracture toughness for this material. As such, failure was not due to classical Mode I failure, but rather due to *K* exceeding what we will term *K_IL_*. Despite the unusual failures, it was found that, up to the point where this phenomenon occurred, the crack growth versus cycles histories could be reasonably accurately predicted using the small crack growth equation developed by the authors in a prior study on AA 7085-T7452 specimens with a fastener hole.

## 1. Introduction

The aluminium alloy 7085-T7452 is a lightweight, high-performance material with contemporary civilian and military aircraft structural applications [1,2,3,4,5,6,7,8,9,10,11]. Its chemical composition (wt%) is 7.5Zn − 1.6Cu − 1.5Mg − 0.12Zr − 0.06Fe − 0.02Si with the rest being Al. The T7452 heat treatment consists of a 6 hr solution treatment at 470 °C, then 5% cold compression, followed by a two-step ageing treatment at 120 °C for 6 h and 160 °C for 10 h. Its mechanical properties are given in Table 1.

It is also one of a number of modern aluminium alloys that the European Aviation Safety Agency (EASA) advises are susceptible to environmentally assisted cracking (EAC), a type of intergranular cracking [2].

**Figure 1:**
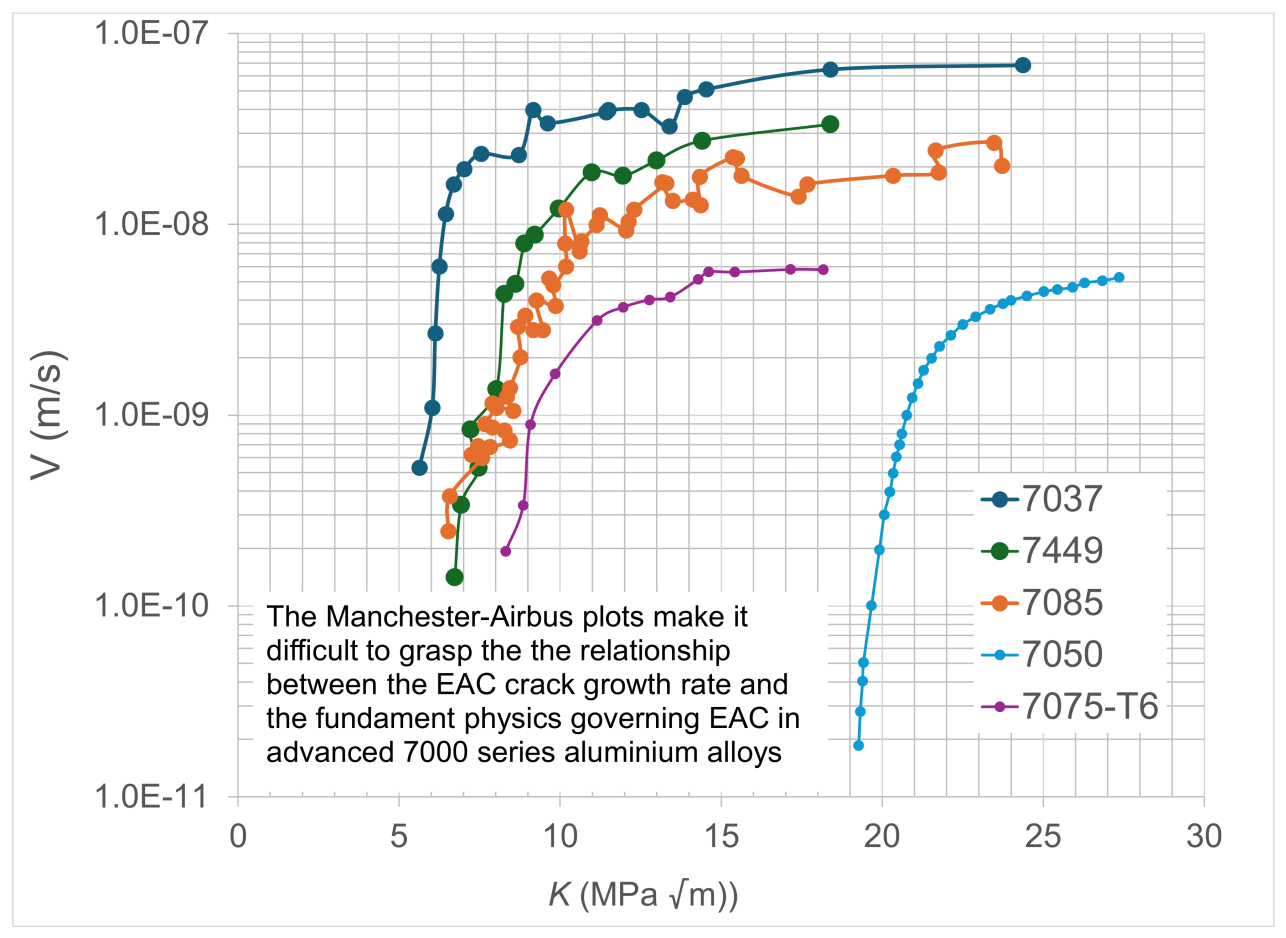
The *K* versus V curves associated with EAC in the aluminium alloys 7037-T7452, 7449-T7651, 7085-T7452, 7050-T7651, and 7075-T6.

As a result of the interest in EAC in AA 7085, which is aptly illustrated in [3,4,5,6,7,8,9,10,11,12,13], the paper by Schwarzenböck et al. [3] suggested that the EAC thresholds for AA 7085, which were determined using double cantilever beam (DCB) specimens at 70 °C and 85% relative humidity (RH), were in the range 6–7 MPa √m; see Figure 1. Schwarzenböck et al. [3] also presented the curves associated with EAC in a range of 7000 series aluminium alloys, viz: 7037-T7452, 7449-T7651, 7085-T7452, and 7050-T7651. The *K* versus crack velocity (*V*) curves determined in this study are shown in Figure 1 together with the EAC curve given in [14] for the aluminium alloy 7075-T6. Here, *K* is the crack tip stress intensity factor and *V* = *da*/*dt*, where *a* is the crack length and *t* is time.

That said, it should also be noted that the seminal paper by Venkateswara Rao, Yu, and Ritchie [15] highlighted that the ranking of the fatigue performance of materials based on tests performed on long cracks does not necessarily correspond to the ranking associated with the growth of small fatigue cracks. Indeed, Gangloff’s subsequent review [16] revealed that the growth of EAC can also be strongly dependent on the size of the initial material defect with short cracks (which [16] defined as being less than 1 mm) having significantly greater EAC crack growth rates than EAC tests performed using ASTM standard compact tension (CT) specimens. The importance of assessing EAC via tests on small naturally occurring cracks is echoed in the papers by Wu et al. [17] and Turnbull [18].

On the other hand, the seminal study by Suresh and Ritchie [19] revealed that true corrosion fatigue can occur at stress intensity levels beneath *K_Iscc_*. (Here, the term *K_Iscc_* refers to the threshold stress intensity factor associated with stress corrosion cracking. In other words, it is the value of *K_Iscc_* that corresponds to a crack growth rate (*da*/*dt*) of 10^−10^ m/s.)

The statement that true corrosion fatigue can occur at stress intensity levels beneath *K_Iscc_* is aptly illustrated by the experimental data contained in the paper by Gangloff [16]. Suresh and Ritchie [19] also explained that, unlike in tests to determine *K_Iscc_*, this phenomenon arises as a result of the crack tip remaining sharp under cyclic fatigue loading, thereby enabling the environment to continuously interact with fresh material.

Returning to the effect of the environment on cracking in operational aircraft, it should be recalled that

(i) The United States Air Force (USAF) Durability Design Hand Book [20], the USAF approach to assessing the risk of failure [21], the Royal Australian Air Force (RAAF) experience with the F/A-18 Classic Hornet Fleet [22], and the investigation into the in-flight failure of a RAAF Macchi jet trainer [23] all note that, to a first approximation, crack growth in operational aircraft starts from the day the airframe enters service.
(ii) The documented deleterious effects of corrosion and the environment on metallic airframes [24,25,26,27,28] when taken in conjunction with point (i) above suggests that the value of Δ*K* at which cracks grow in an operational aircraft is very low. This conclusion is consistent with the statement contained in Appendix X3 of the fatigue test standard ASTM E647 [29] that it is unclear if naturally occurring cracks actually have a fatigue threshold.

As such, it is suggested that the EAC rankings implied in the paper by Schwarzenböck [3] may not necessarily reflect the rankings for an aircraft that is operated in an aggressive environment. Indeed, the question of the relative in-service ranking of aerospace aluminium alloys is made more interesting as a result of the findings echoed in [30,31,32,33,34,35] that crack growth in civil transport and combat aircraft often decouples with corrosion and EAC occurring on the ground and crack growth occurring at altitudes where corrosion activity is essentially suspended.

In this context it should be noted that, since it has been suggested that environmental and fatigue crack growth effects may decouple, the authors of [36] presented the results of an experimental test programme whereby AA 7050-T7451 specimens were first exposed to an aggressive environment to develop corrosion pits that were of the order of 0.1 mm deep. The exposure process used involved placing approximately 50 μL droplets of 0.606 M Sodium Chloride (NaCl) solution on the surface of one face of the coupons at the mid-section using a syringe, making a droplet on the surface. The specimens were then placed in an environmental chamber maintained at 20 °C and 98% relative humidity (RH). At 98% RH, the equilibrium concentration for NaCl solution is 0.606 M, so the droplet would neither spread nor contract due to volumetric changes. The coupons were placed in the humidity chamber for 6 days, following which they were removed and placed in an oven at 50 °C for an hour to dry out the corrosion sites. This was done to replicate pit depths found in RAAF F/A-18 aircraft. The specimens were subsequently subjected to a marker block load spectrum that consisted of 250 cycles at an *R*-ratio of 0.1 and 15,000 cycles at an *R*-ratio of 0.8. The maximum stress in the spectrum was 250 MPa. The fatigue test was halted every 15 marker load blocks and the specimens were then re-exposed to an aggressive environment for a week. This procedure was repeated until the specimens failed. The *R*-ratio is defined as the ratio *K_min_*/*K_max_* where *K_max_* and *K_min_* are the maximum and minimum values of the stress intensity factor (*K*) in a load cycle.

The purpose of repeatedly halting the fatigue testing and exposing the specimen was twofold. One reason was to attempt to mimic, to a limited extent, the effect of combined intermittent on-ground environmental exposure and flight loads at altitudes where the temperature was such that corrosion did not occur. The second purpose stated in [36] was to ensure that any crack tip blunting or change in crack path would be noticed in the constant amplitude growth and rightly attributed to the interaction between corrosion and fatigue.

One of the key outcomes of this study was the fact that the fatigue threshold associated with the fastest growing crack, that nucleated from the corrosion pits, was very low (approximately 0.1 MPa √m). This finding supports the conclusion reached by Gangloff [16] that the fatigue threshold associated with the growth of EAC can be strongly dependent on the size of the initial material defect. Since this value is significantly beneath the value of *K_Iscc_* shown in Figure 1 for 7050-T7451, it also raises questions concerning the EAC rankings obtained using DCB and CT specimens.

Consequently, given the unexpected sensitivity of AA 7085-T7452 to EAC, the importance of understanding the growth of small naturally occurring three dimensional cracks that nucleate from surface corrosion/pitting, and the prior tests [36] on AA 7050-T7451 when pre-exposed to an aggressive environment and subsequently subjected to periods of repeated fatigue loads and repeated periods of further environmental exposure, the present paper focuses attention on the following two questions:

(a) Do small naturally occurring three dimensional (3D) cracks that nucleate in AA 7085-T7452 from corrosion pits, that have resulted from prolonged exposure to an aggressive environment, exhibit any other unusual fatigue failure mechanism?
(b) If the answer to this question is yes, then can the crack growth equation developed by the authors in [1] for the growth of small naturally occurring 3D cracks in AA 7085-T7452 be used to compute the crack growth histories?

It should be stressed that, given the widespread use of AA 7085-T7452 in both civil transport and military aircraft, the focus of this study is not the underlying materials science but rather the fracture mechanics and its implications with respect to the structural integrity and the airworthiness of aircraft structures that are fabricated from AA 7085-T7452.

## 2. Materials and Methods

### 2.1. Specimen Geometry

**Figure 2:**
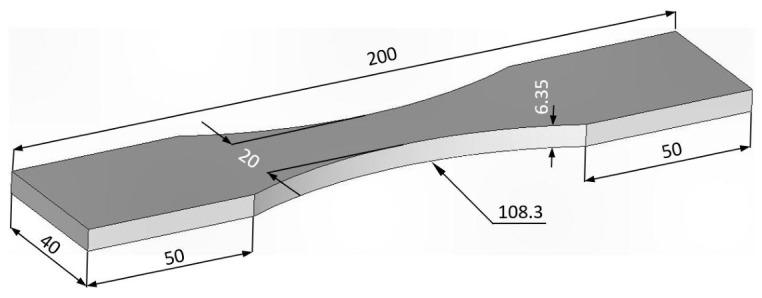
A schematic diagram of the test specimens.

The specimen geometry used to study the growth of small naturally occurring cracks in these two AA 7085-T7452 specimens, which were labelled 7085_1 and 7085_4, is shown in Figure 2. As in [1], the specimens were machined from an AMS 4414 compliant AA 7085-T7452 die forging in the T-L orientation. Furthermore, as in [1], these specimens were also exposed for 14 days to an ASTM B117-19 Standard [37] 5% NaCl salt fog environment at 35 °C. Indeed, all of the specimens tested in [1] and in the present paper were exposed at the same time and in the same ASTM B117-19 environmental chamber. The reason for limiting the exposure to fourteen days was that, as explained in [1], at this stage, the level of corrosion was particularly severe; see [1] for details.

The reason for adopting this particular approach was twofold. The first reason was that it enables a direct comparison with prior studies [1]. The second reason was that, as outlined above, experience suggests [30,31,32,33,34,35] that, for operational aircraft, the problem space often decouples with corrosion and EAC occurring on the ground and crack growth occurring at altitudes where corrosion activity is essentially suspended.

As a result of these observations, the role of exposure in the B117-19 environmental chamber was to create a severe level of corrosion pitting. This approach has the added advantage that, as is also explained in [1], it exceeded the extent of corrosion pitting seen after twelve months of outdoor exposure to a hot–humid marine environment on Hainan Island, China [38]. Here, the average air temperature, relative humidity, and Cl- deposition rates were approximately 23.9 °C, 87.6%, and 14.6 mg/(m^2^·d), respectively. Furthermore, the corrosion depths were comparable to the depths reported in [6] for AA7085-T7452 specimens that had experienced twelve months of exposure at RAAF Base Williamtown in New South Wales (Australia) and with the corrosion pit depths reported in [36] for AA 7050-T7451.

### 2.2. The Fatigue Load Spectrum

Once the specimens had been removed from the environmental chamber they were cleaned and dried to remove any contaminants. The process used was as follows: The specimens were first washed with clean water. They were then cleaned with isopropyl alcohol for 15 min and subsequently air dried.

The specimens were then fatigue tested, in a laboratory air environment, in a 100 kN servo-hydraulic fatigue test machine under load control. The marker block load spectrum applied to these specimens consisted of: 15,000 cycles at *R* = 0.8 and 300 cycles at *R* = 0.1. The maximum load P_max_ in each load block was held constant at 50.8 kN. The test frequency was 5 Hz. The maximum stress in the central working section was approximately 400 MPa. The difference between the load spectra used in the current tests and that used in [1] is that the maximum load used in [1] was 32 kN. This load level corresponded to a stress in the central region of approximately 250 MPa.

As in [1], the marker block load spectrum enabled the crack growth histories to be determined by quantitative fractography.

### 2.3. Computing the Crack Growth Histories

The paper by Peng et al. [1] revealed that the growth of small cracks in the previous test programme, which also studied fatigue crack growth (FCG) in AA 7085-T7452 specimens that, prior to fatigue testing, had been exposed in an ASTM B117-19 environmental chamber, could be predicted using the crack growth equation

*da*/*dN* = 1.2 × 10^−10^ [(∆*K* − 0.1)/√(1 − *K*_max_/65)]^2^ (1)

Here, ∆*K* = *K_max_* − *K_min_*, where *K_max_* and *K_min_* are the maximum and minimum values of the stress intensity factor (*K*) in a cycle. Consequently, Equation (1) was used to predict the small crack growth histories in the current AA 7085-T7452 fatigue tests.

The approach used to predict the crack growth histories is the same as that used in [1,35,39,40,41,42,43,44]. A computer-aided design (CAD) model for the specimen is first created. The CAD model is then used to develop a finite element model of the uncracked structure. As in [1,35,36,39,40,41,42,43,44], for the assumed initial crack shape, the stress intensity factor distribution around the crack is computed using the 3D finite element alternating method FEAT; see [45,46,47,48,49,50]. An advantage of using the FEAT to compute the stress intensity factors for 3D cracks is that it is only necessary to use a finite element model of the uncracked structure; see [45,46,47,48,49,50]. (This approach was also used in [1,35,39,40,41,42,43,44]). An alternative approach would have been to use the Zencrack^®^ software interface [51] with the general-purpose finite element programmes Abaqus^®^, NASTRAN^®^, and ANSYS^®^. Examples of this approach can be found in [52,53,54,55,56].

Having determined the stress intensity factors, Equation (1) is then used to predict the increment in the crack shape. Armed with this information, a new updated crack shape is determined and the process is repeated. This approach has been used to study crack growth in a range of operational aircraft under representative operational loads [35,36,39,49] as well as crack growth in rail freight vehicles under representative operational load spectra [44].

This approach has also been used for computing the growth of naturally occurring 3D cracks in a range of both conventionally and additively manufactured (AM) materials [1,35,36,39,40,41,42,43,44,47,49].

#### 2.3.1. Computing the Crack Growth History Associated with Specimen 7085_1

**Figure 3:**
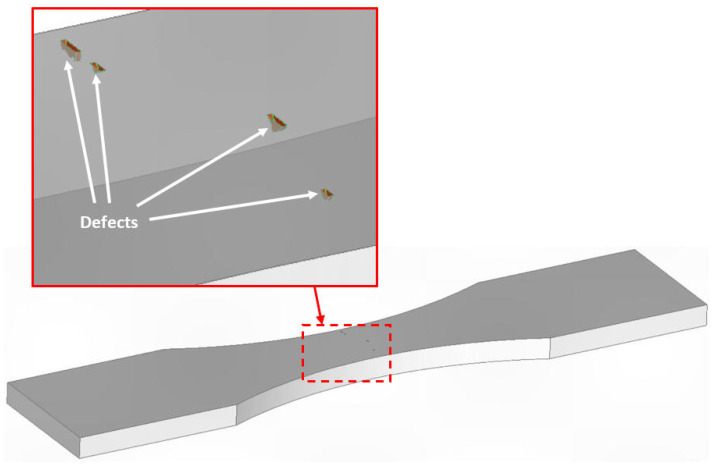
CAD model used to develop the finite element analysis for Specimen 7085_1.

**Figure 4:**
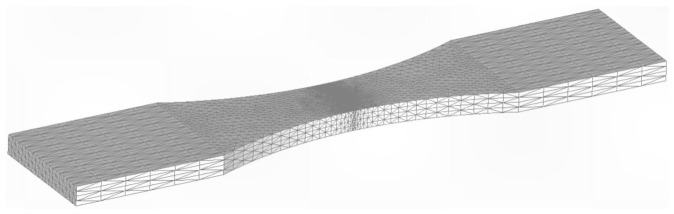
Mesh 1: 52,721 ten-noded iso-parametric tetrahedral elements and 80,043 nodes.

**Figure 5:**
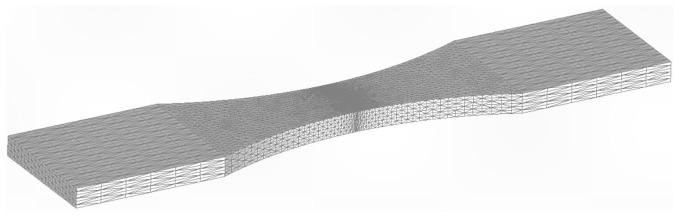
Mesh 2: 102,059 ten-noded iso-parametric tetrahedral elements and 153,525 nodes.

**Figure 6:**
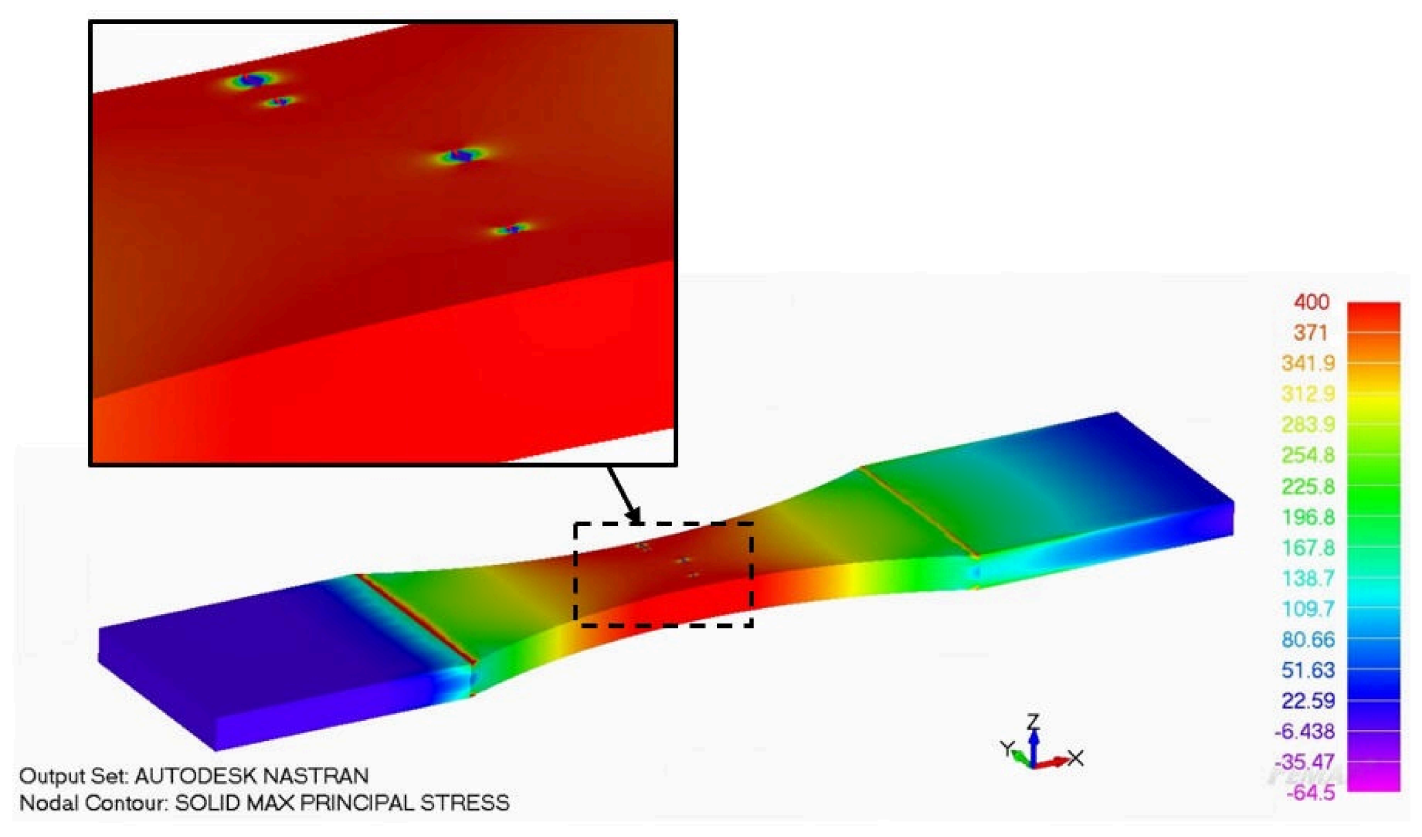
The maximum principal stress for Specimen 7085_1 at a remote load of 50.8 kN; the stress units are in MPa. The stress field at the locations where the crack(s) nucleated in the two analyses differed by less than 1.7%.

The CAD model developed for Specimen 7085_1 is shown in Figure 3. This CAD model, which accounted for the corrosion pitting from which cracks grew, was used to develop two different finite element meshes of the uncracked specimen. (The need to model the material discontinuity from which the cracks nucleated is highlighted in [43]. If this is not done, then the stress intensity factor solutions associated with small three-dimensional cracks that have nucleated from the feature can be greatly overestimated; see [43] for details.) The first mesh, which was a coarse mesh, had 52,721 ten-noded iso-parametric tetrahedral elements and 80,043 nodes; see Figure 4. The second mesh, which was a finer mesh, had 102,059 ten-noded iso-parametric tetrahedral elements and 153,525 nodes; see Figure 5. The stress field at the locations where the cracks nucleated in these two analyses differed by less than 1.7%. The stress field associated with the fine mesh is shown in Figure 6. The crack growth analysis used the stress intensity solutions determined using the fine mesh in conjunction with Equation (1).

#### 2.3.2. Computing the Crack Growth History Associated with Specimen 7085_4

**Figure 7:**
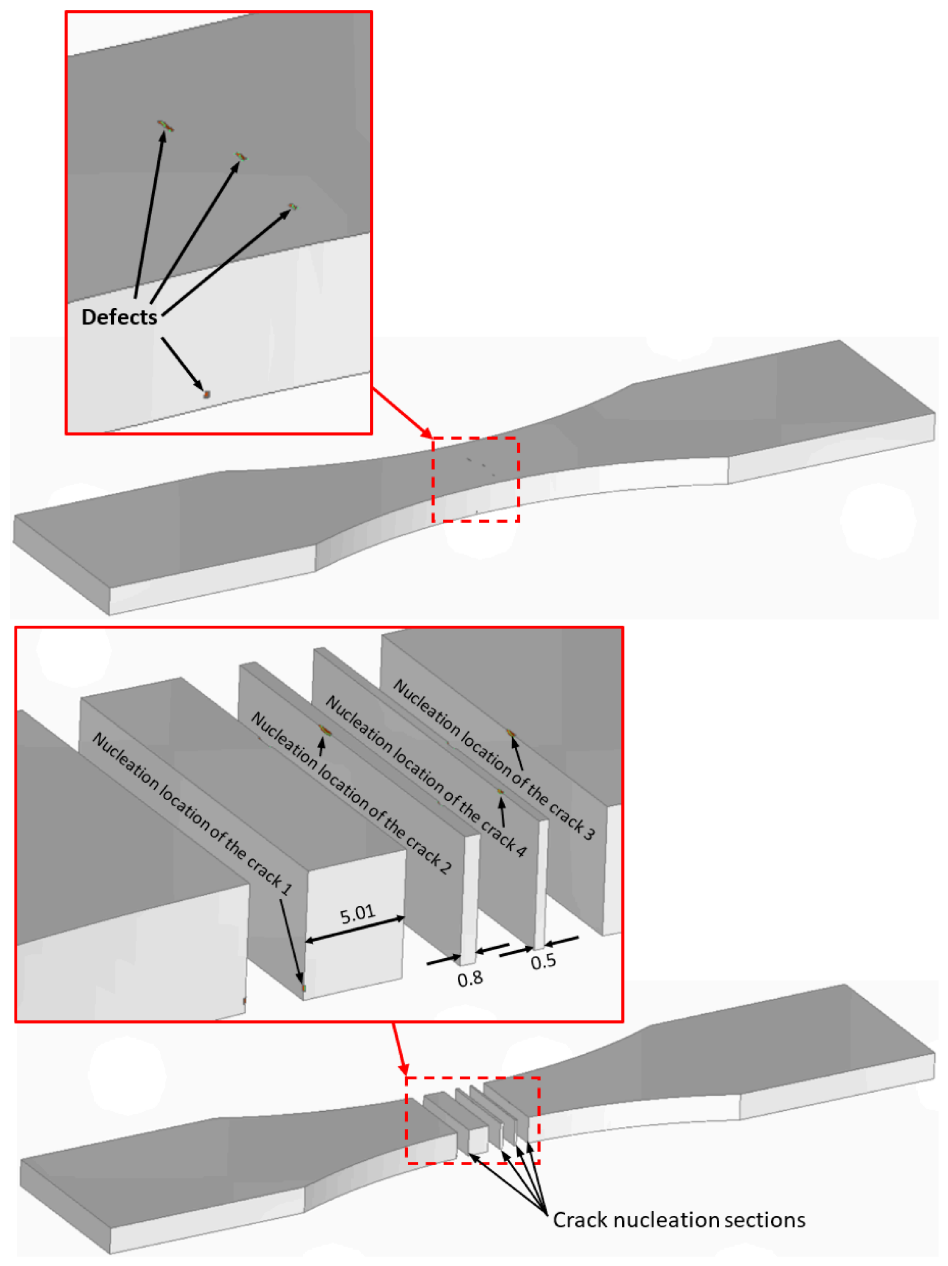
CAD model used to develop the finite element analysis for Specimen 7085_4.

**Figure 8:**
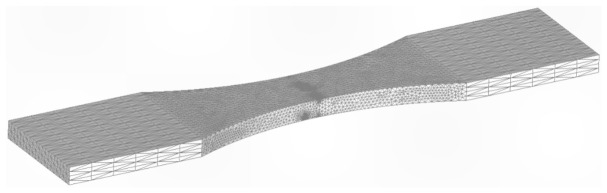
Mesh 1: 76,168 ten-noded iso-parametric tetrahedral elements and 115,779 nodes.

**Figure 9:**
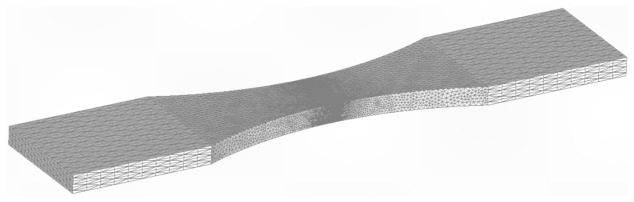
Mesh 2: 178,411 ten-noded iso-parametric tetrahedral elements and 263,168 nodes.

**Figure 10:**
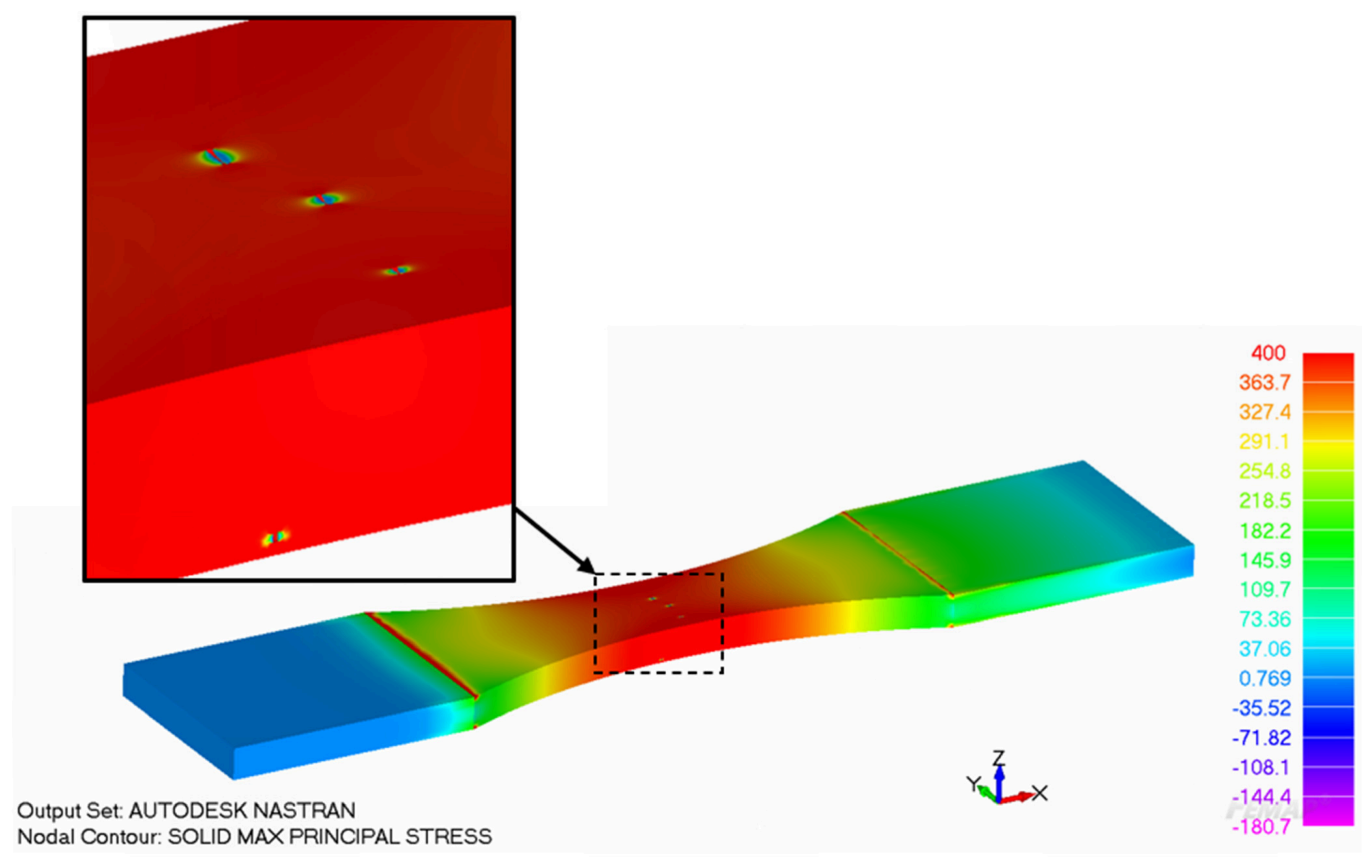
The maximum principal stress for Specimen 7085_4 at a remote load of 50.8 kN; the stress units are in MPa. The stress field at the locations where the crack(s) nucleated in the two analyses differed by less than 1.5%.

The CAD model developed for Specimen 7085_4 is shown in Figure 7. This CAD model, which also accounted for the corrosion pitting from which cracks grew, was more complex than that used in the prior study. This was due to the fact that cracking developed from corrosion pits that lay on a number of different planes; see Figure 7. This CAD model was also used to develop two different finite element meshes of the uncracked specimen. The first, a coarse mesh, had 76,168 ten-noded iso-parametric tetrahedral elements and 115,779 nodes; see Figure 8. The second, which was a finer mesh, had 178,411 ten-noded iso-parametric tetrahedral elements and 263,168 nodes; see Figure 9. The stress field associated with the fine mesh is shown in Figure 10. The stress field at the locations where the cracks nucleated in these two analyses differed by less than 1.5%. The crack growth analysis used stress intensity solutions determined using the fine mesh in conjunction with Equation (1).

## 3. Experimental Results

### 3.1. Effect of Prior Exposure

**Figure 11:**
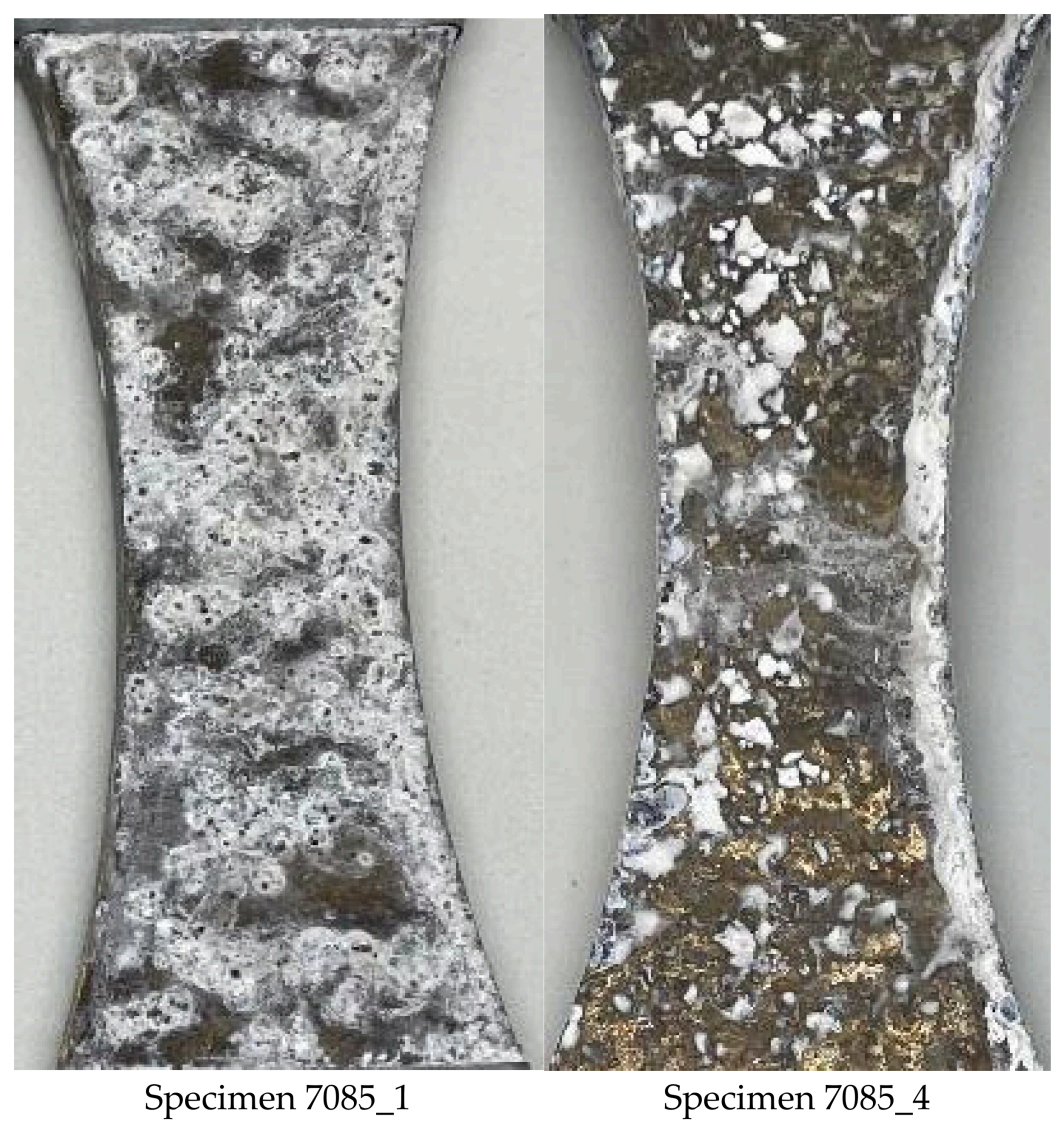
Photographs of the extensive corrosion seen on specimens 7085_1 and 7085_4 after fourteen days of exposure to a 5% NaCl salt fog at 35 °C.

Pictures of the extensive corrosion seen on specimens 7085_1 and 7085_4 after fourteen days in the environmental chamber are given in Figure 11. As would be expected, since these specimens were exposed at the same time as the specimens discussed in [1], the level of corrosion seen in these specimens was similar to that detailed in [1]. The precise depths of the corrosion pits, from which cracks nucleated, are shown in the next section.

### 3.2. The Fatigue Tests

#### 3.2.1. Specimen 7085_1

**Figure 12:**
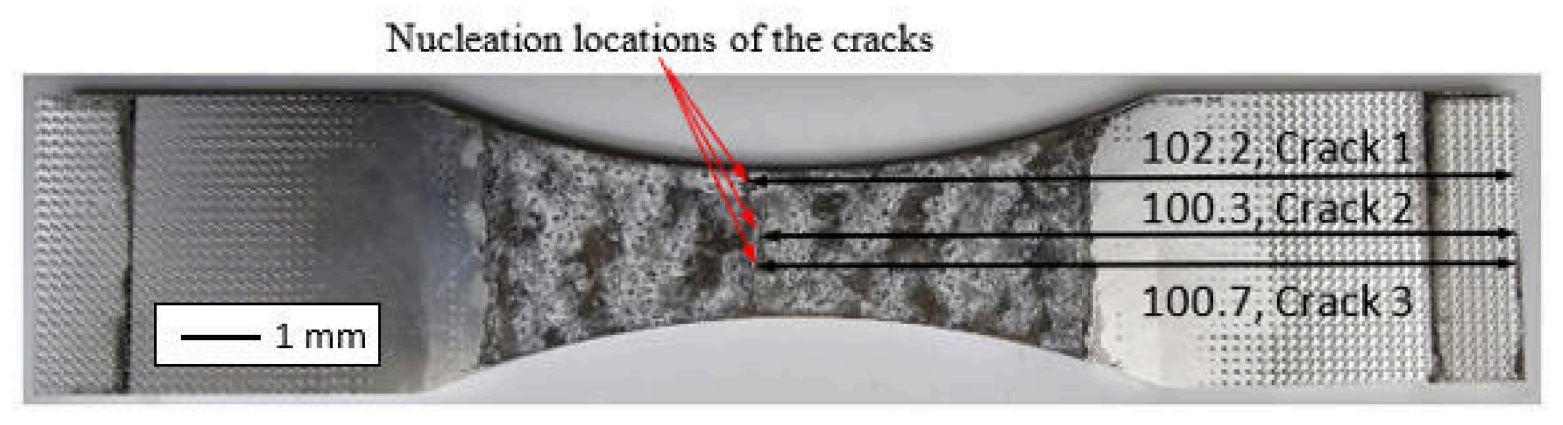
Specimen 7085_1 after test (the unit of measurement used in the figure is “mm”).

**Figure 13:**
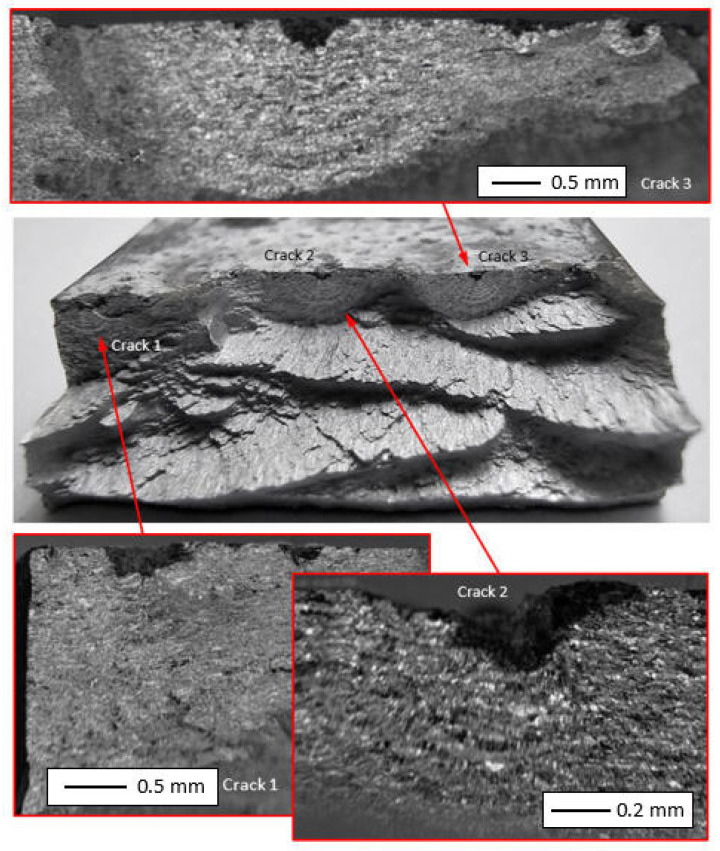
The failure surface and pit depths associated with Specimen 7085_1 (the unit of measurement used in the figure is “mm”).

**Figure 14:**
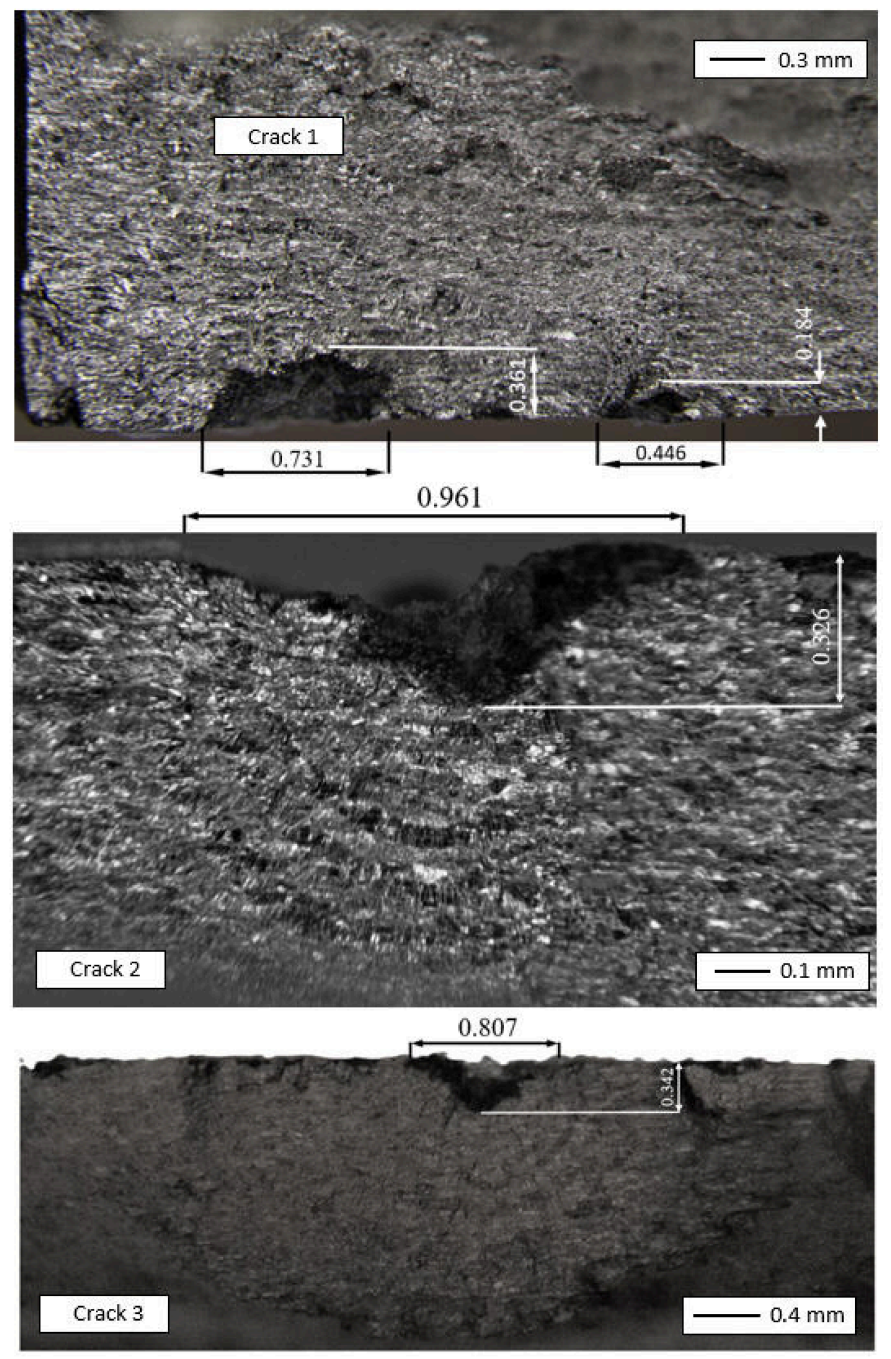

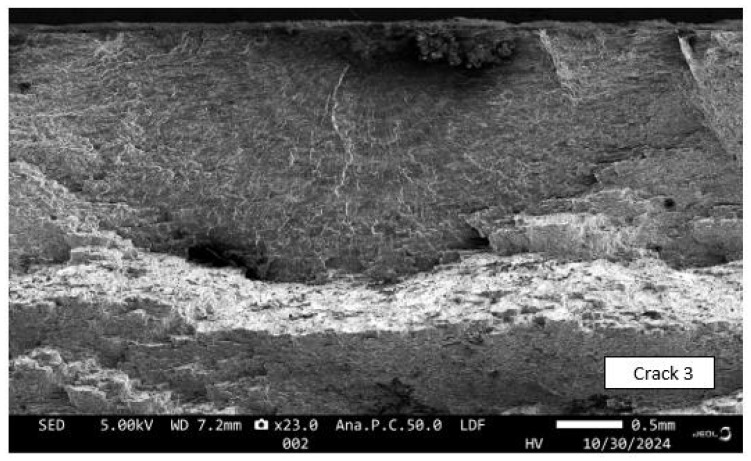
Dimensions of the corrosion pits associated with the three nucleated cracks in Specimen 7085_1 (the unit of measurement used in the figure is “mm”).

Specimen 7085_1, which was tested first, failed after 167,226 cycles. This equates to approximately 10.93 marker blocks. A plan view of the failed specimen is shown in Figure 12. Here we see three locations where cracking nucleated. As can be seen in Figure 13, these three cracks, which nucleated at corrosion pits, were on slightly different planes. The dimensions associated with the corrosion pits from which each of these three cracks nucleated are shown in Figure 14. These pit depths are again consistent with those shown in the prior study [1] into the effect of pre-exposure on AA7085-T7452 specimens that were tested at 250 MPa. This is to be expected, since the specimens tested in this study and in [1] were simultaneously exposed in the same B117-19 environmental chamber. The pit depths are also consistent with those given in [36] for pre-exposed AA 7050-T7451 specimens.

**Figure 15:**
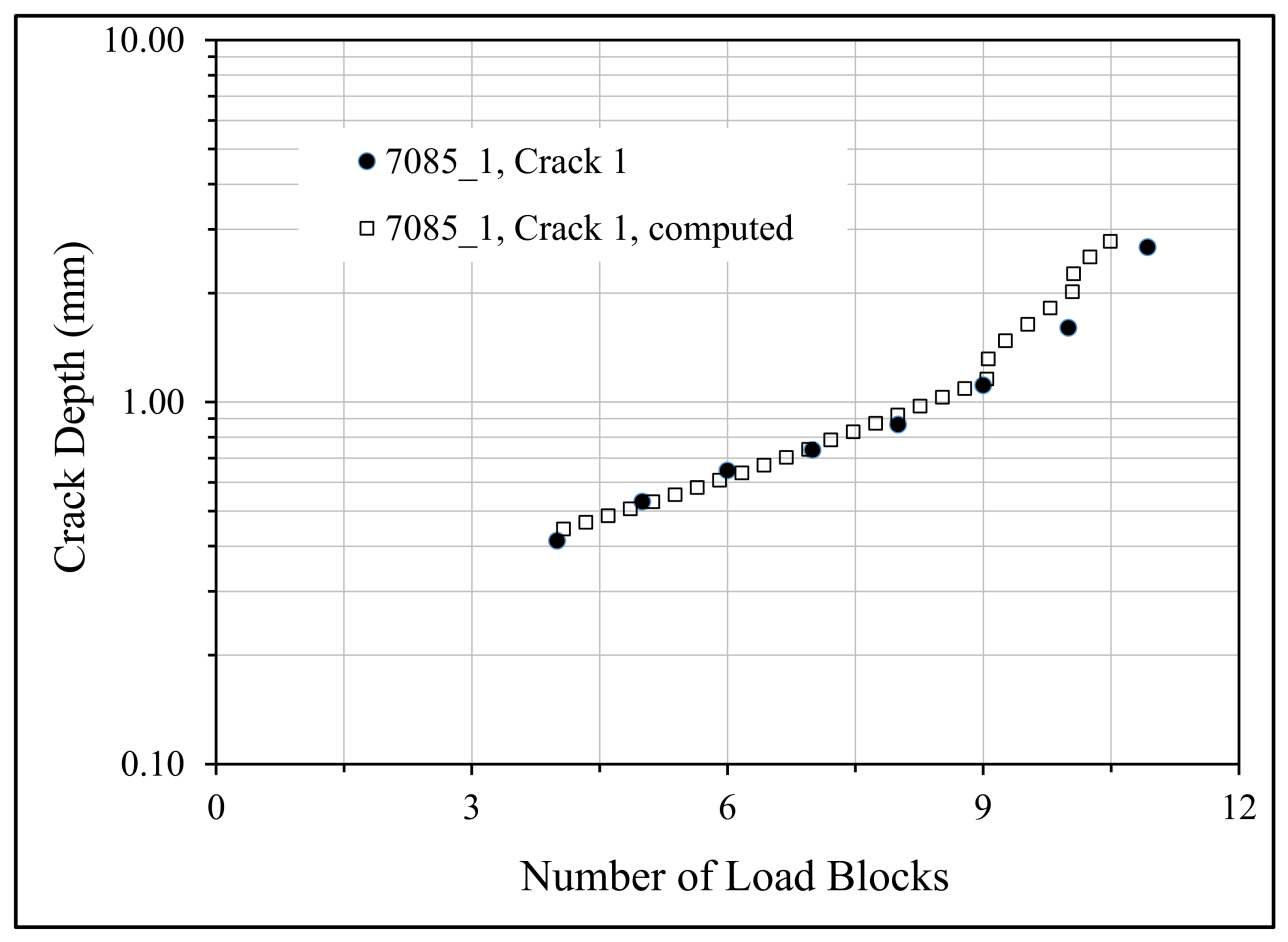
The measured and computed curves associated with the Crack 1 in Specimen 7085_1.

**Figure 16:**
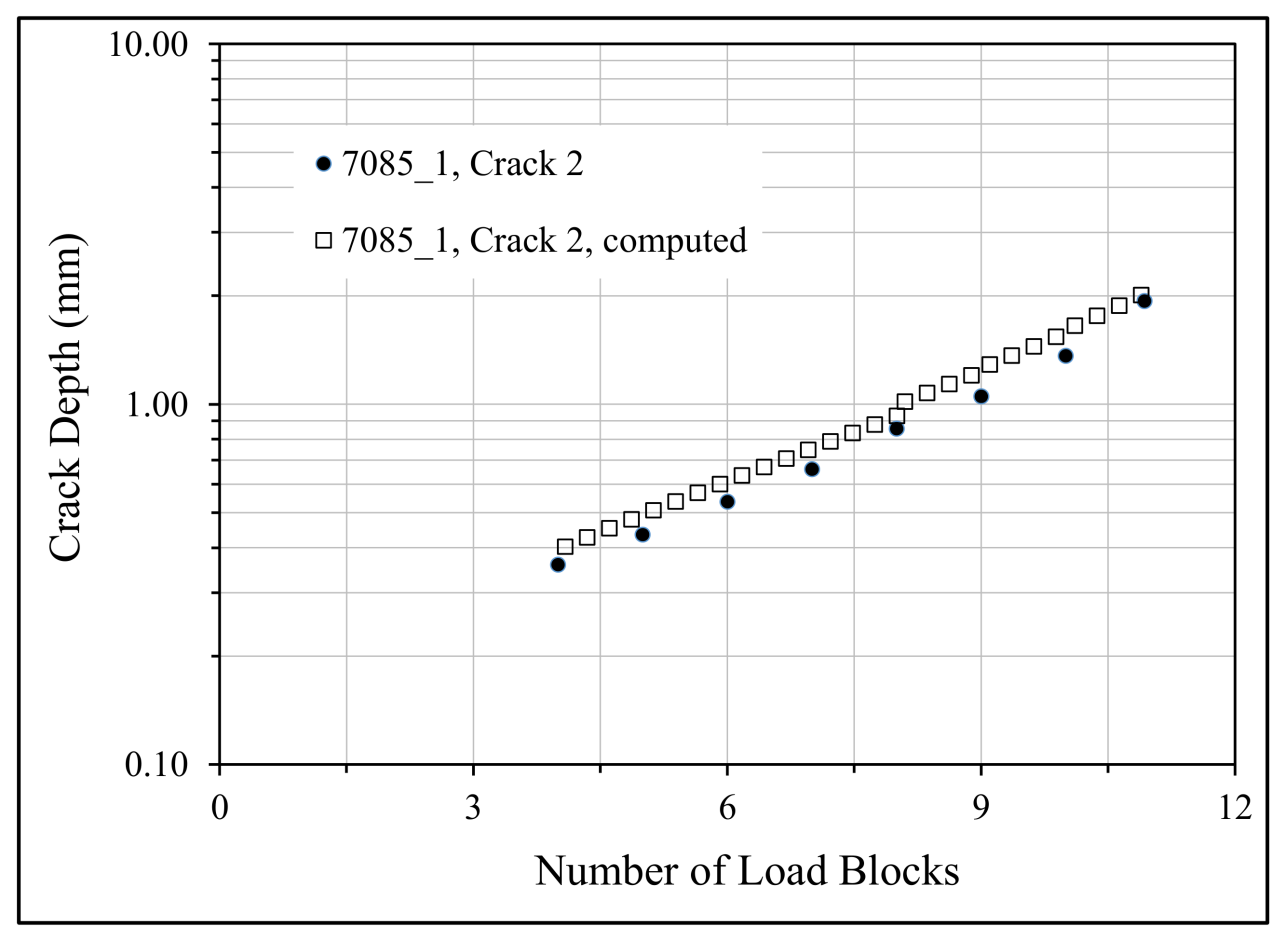
The measured and computed curves associated with the Crack 2 in Specimen 7085_1.

**Figure 17:**
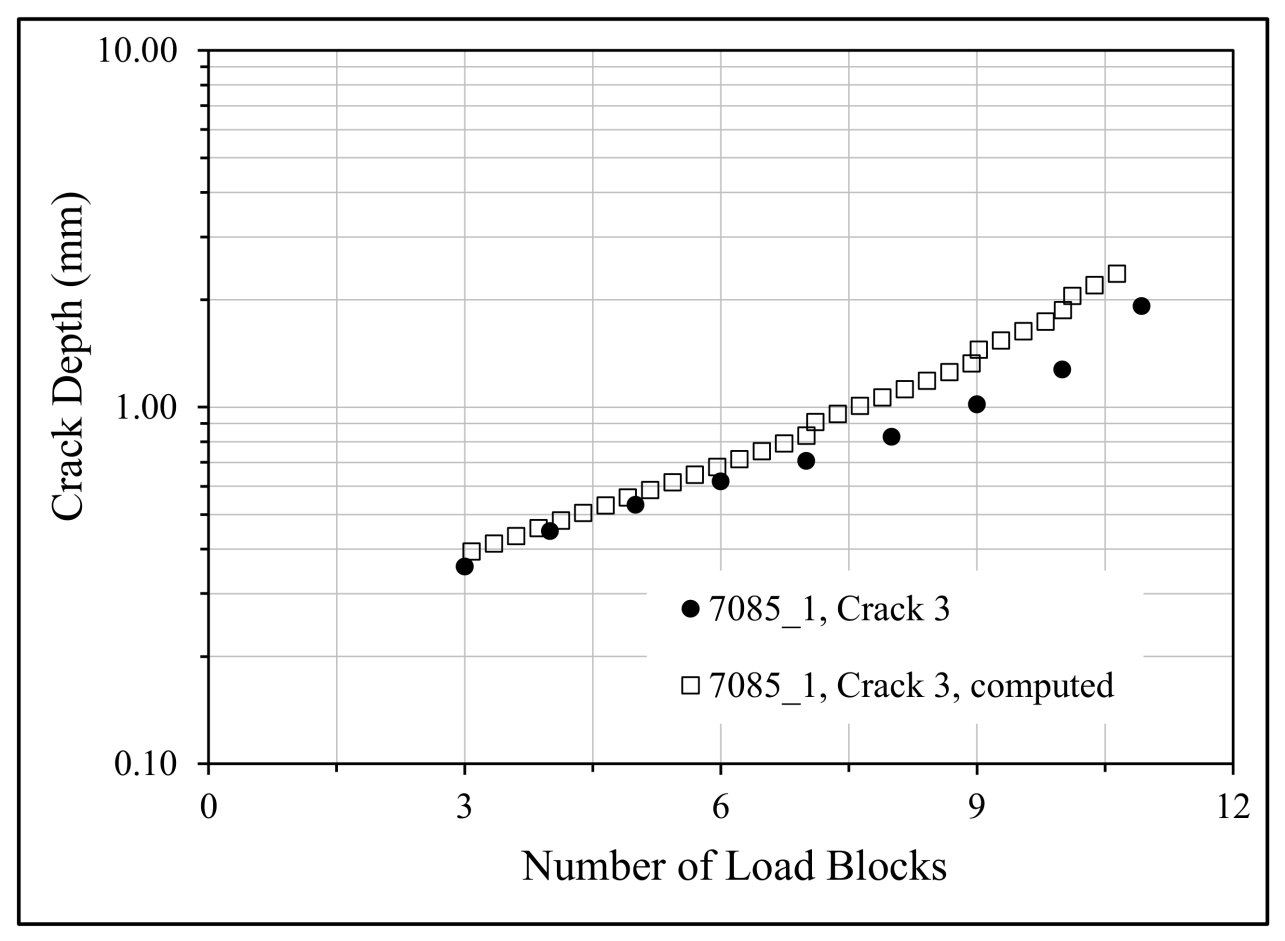
The measured and computed curves associated with the Crack 3 in Specimen 7085_1.

As can be seen in Figure 13 and Figure 14, the failure surface had a most unusual appearance. Whilst there were three well defined cracks (see Figure 13), once the crack had reached a depth of approximately 2 mm, the crack turned to be at a near 90° angle to the initial Mode I fracture plane and final failure resulted in what, for want of a better description, we will term a layered failure appearance. Figure 14 presents an SEM image that aptly illustrates this phenomenon. The measured crack growth histories, up to the point where the cracks transition from a classical Mode I crack to this layered-like failure surface, are shown in Figure 15, Figure 16 and Figure 17. It should be stressed that this failure mechanism has not previously been reported.

Figure 13 reveals that failure was not a simple Mode I failure. Hence, failure was not due to the value of *K* exceeding its Mode I fracture toughness (*K_I_c*). This latter point will be discussed in more detail in Section 4.

The realisation that this is not a simple Mode I failure has implications with respect to the structural integrity and the airworthiness of aircraft structures that are fabricated from AA 7085-T7452.

#### 3.2.2. Specimen 7085_4

**Figure 18:**
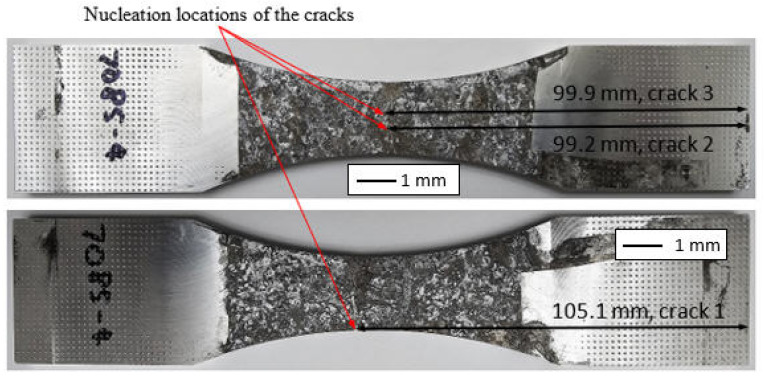
Specimen 7085_4 after test (the unit of measurement used in the figure is “mm”).

**Figure 19:**
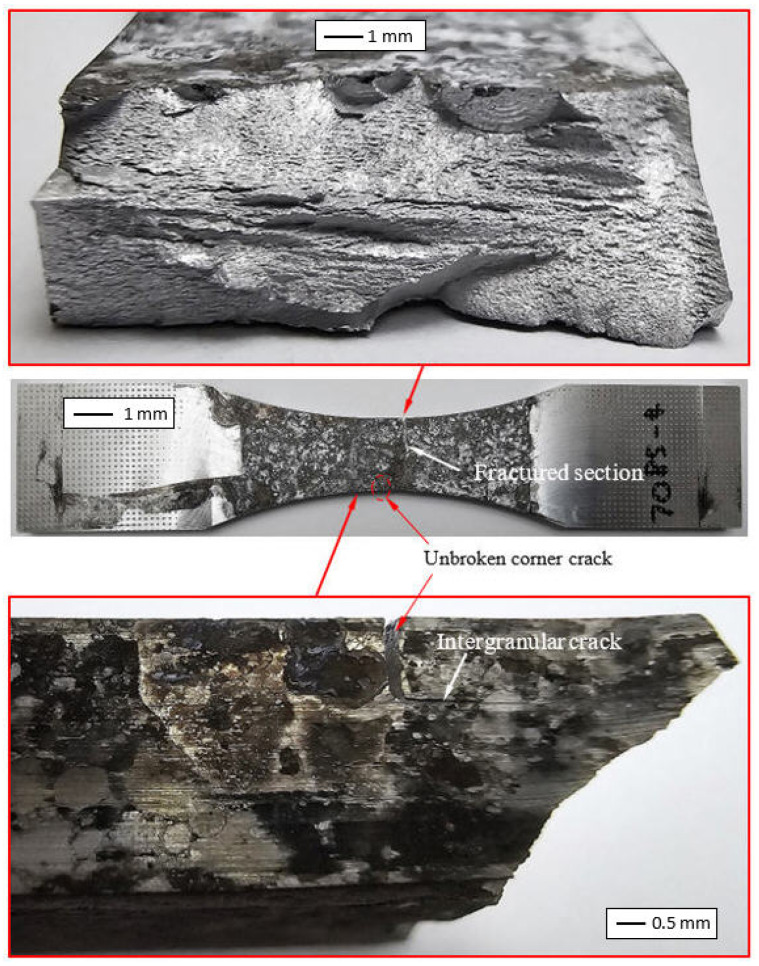
The failure surfaces associated with Specimen 7085_4 (the unit of measurement used in the figure is “mm”).

**Figure 20:**
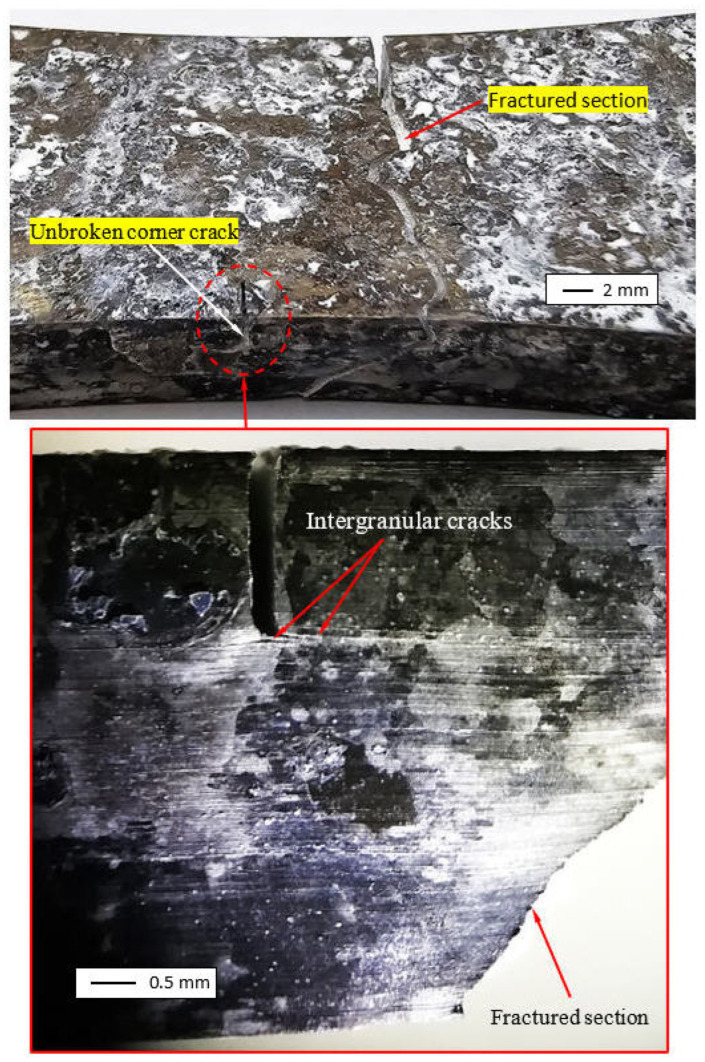
The failure surface associated with Crack 5, which lay some distance from the primary failure plane (the unit of measurement used in the figure is “mm”).

**Figure 21:**
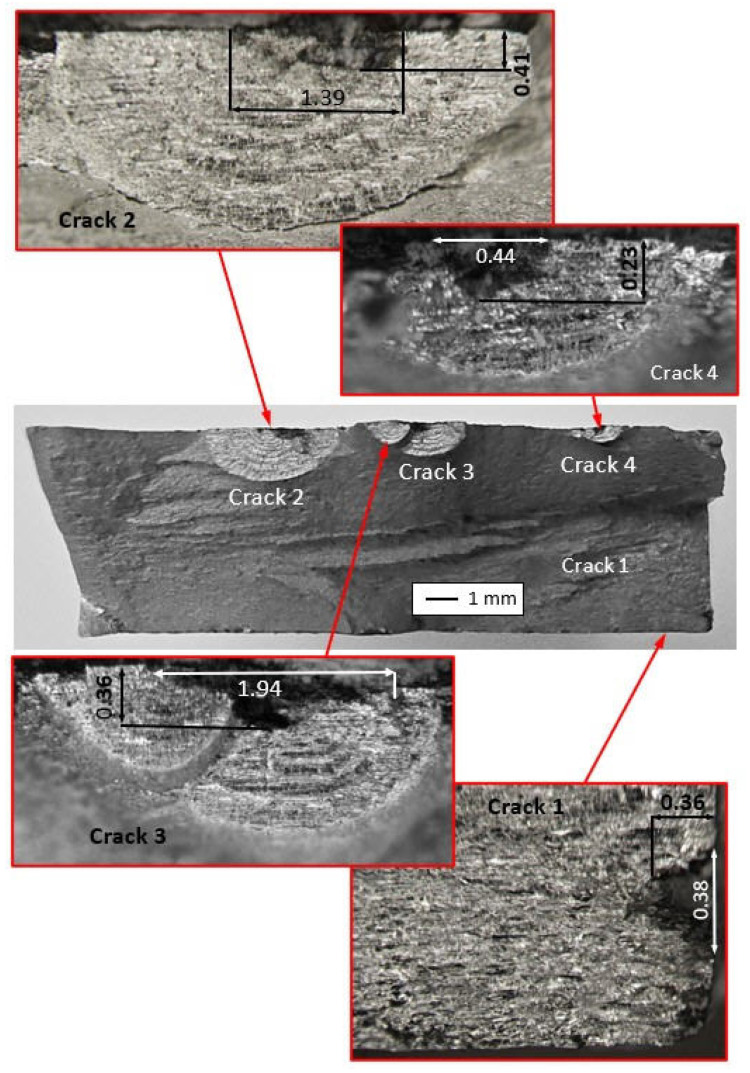
The failure surface and pit depths associated with Specimen 7085_4 (the unit of measurement used in the figure is “mm”).

Specimen 7085_4 failed after 137,938 cycles. This equates to approximately 9.02 marker blocks. A plan view of the failed specimen is shown in Figure 18. At the resolution associated with this figure, only three of the five cracks can be clearly seen, namely Cracks 1, 2 and 3. That said, Figure 19, Figure 20 and Figure 21 show that there were in fact five distinct cracks. Three of these cracks, which are labelled Cracks 2–4, were associated with the primary failure surface. These cracks are shown in Figure 18. Although not as prominent as that shown in Figure 13, the failure surface nevertheless exhibits features that resembled the layered failure appearance shown in Figure 13. Cracks 1 and 5 lay some distance from the failure plane. As can be seen in Figure 20, Crack 5 further highlights the propensity of cracking from corrosion pits in AA 7085-T7452 to first grow as per a classical small Mode I crack, by this we mean normal to the upper surface, before turning to be essentially parallel to the upper surface.

Only those cracks that lay on the primary failure plane, namely Cracks 2, 3, and 4, were analysed. The dimensions associated with the corrosion pits from which these three cracks nucleated are shown in Figure 21. These pit depths are again consistent with those shown in [1], i.e., for the prior study into the effect of pre-exposure on AA7085-T7452 specimens that were tested at 250 MPa.

**Figure 22:**
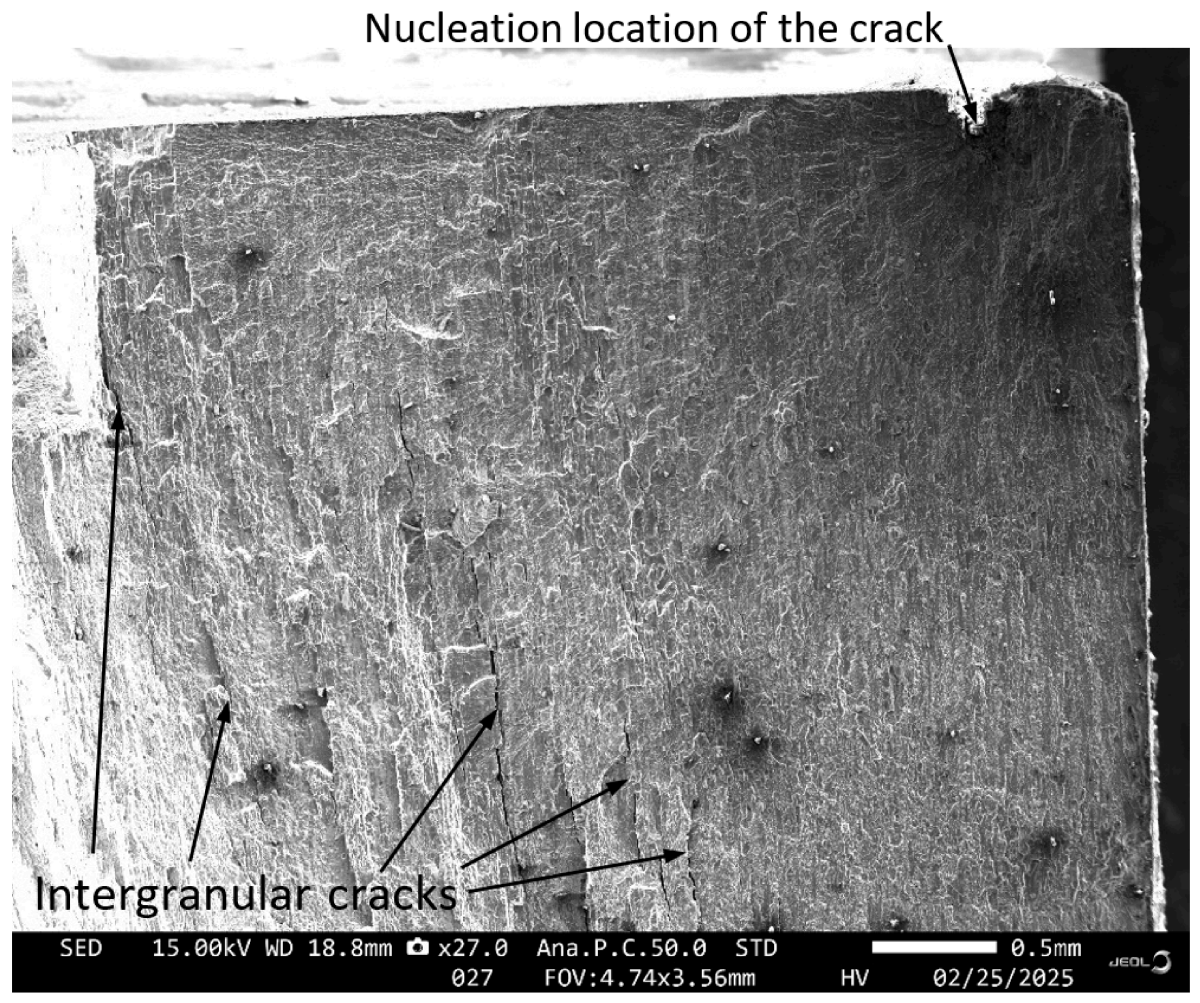
Local details of Crack 1. Note that the orientation of the specimen is at ninety (90) degrees to that shown in Figure 21.

**Figure 23:**
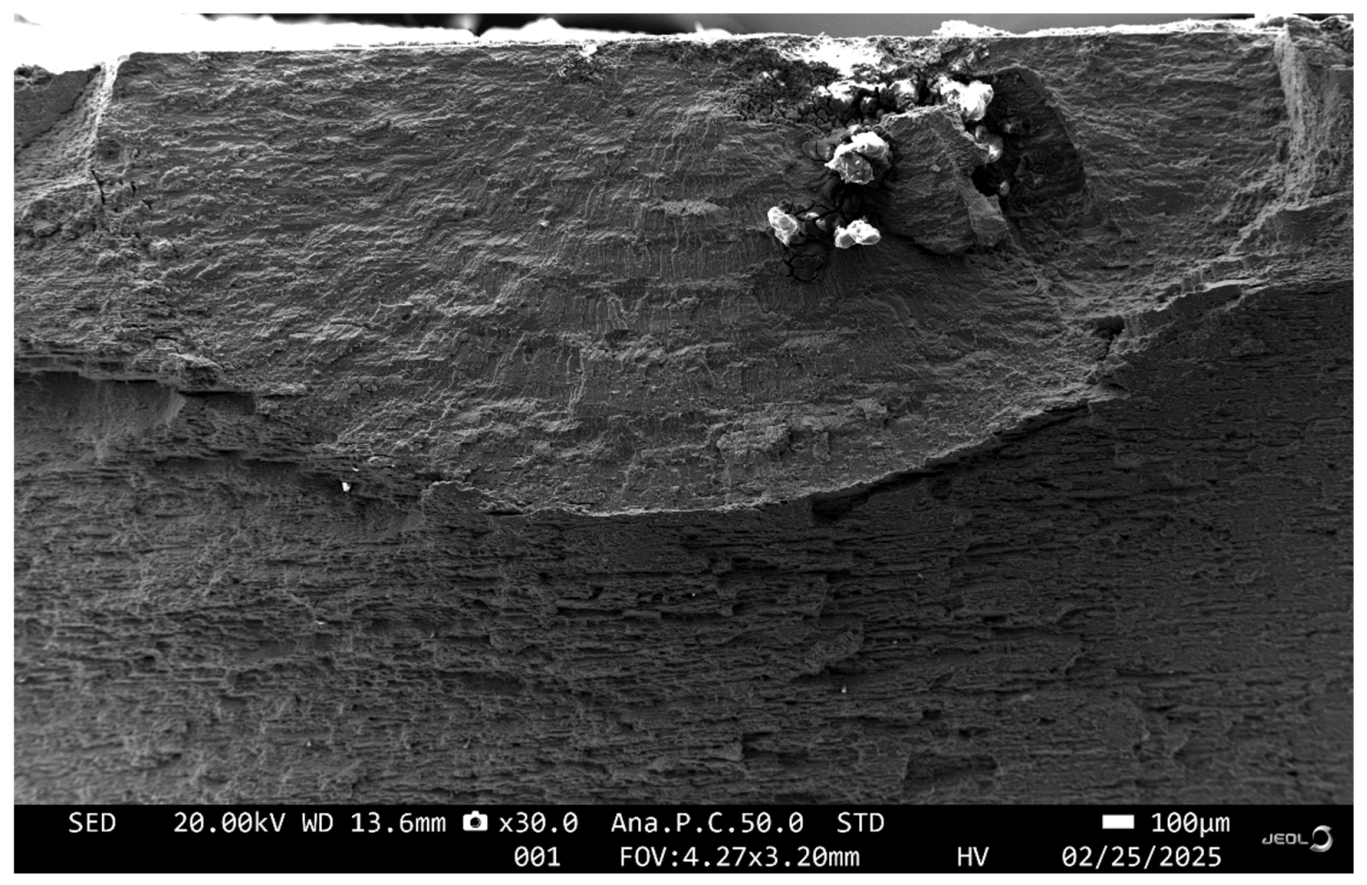
Local details of Crack 2.

**Figure 24:**
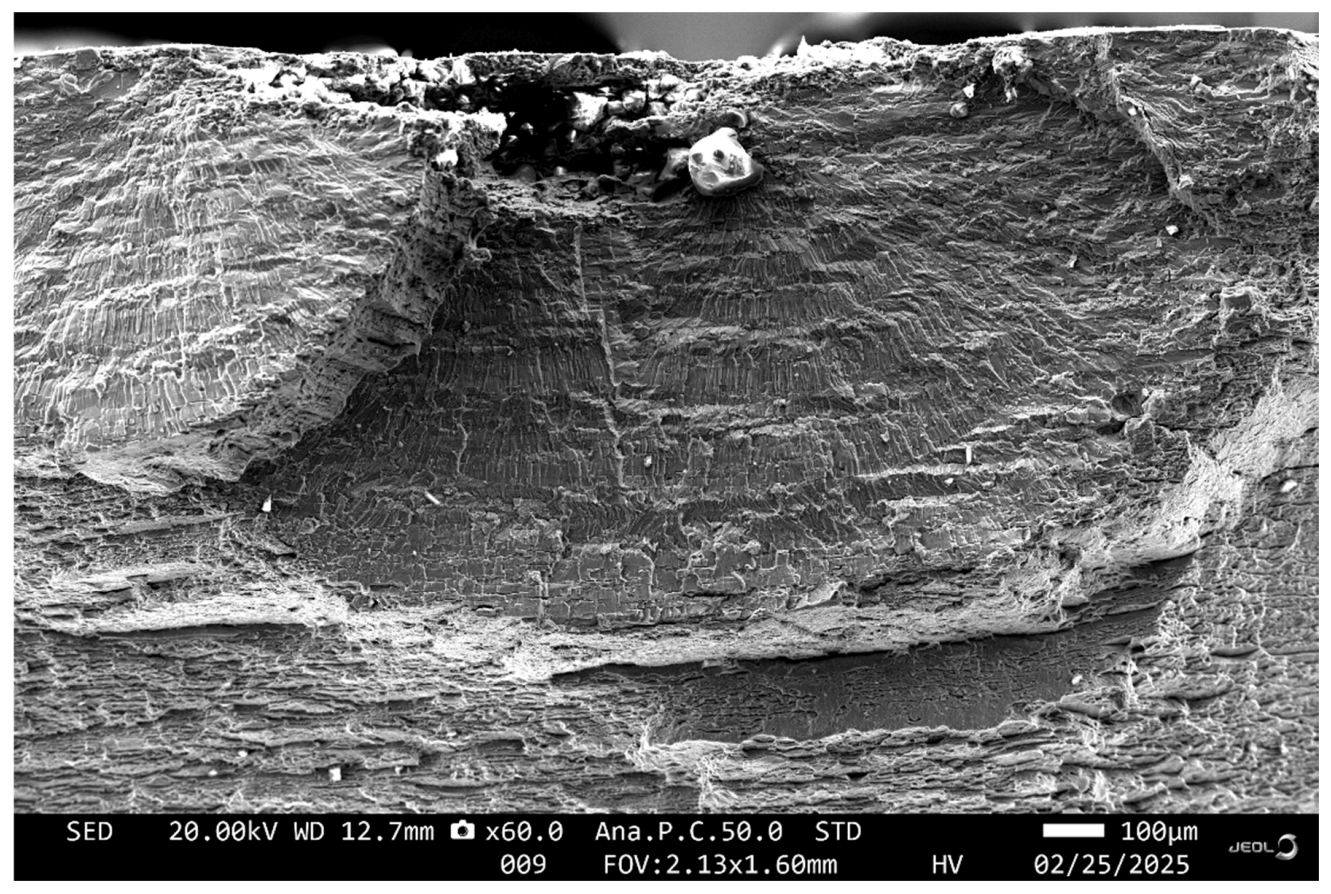
Local details of Crack 3.

**Figure 25:**
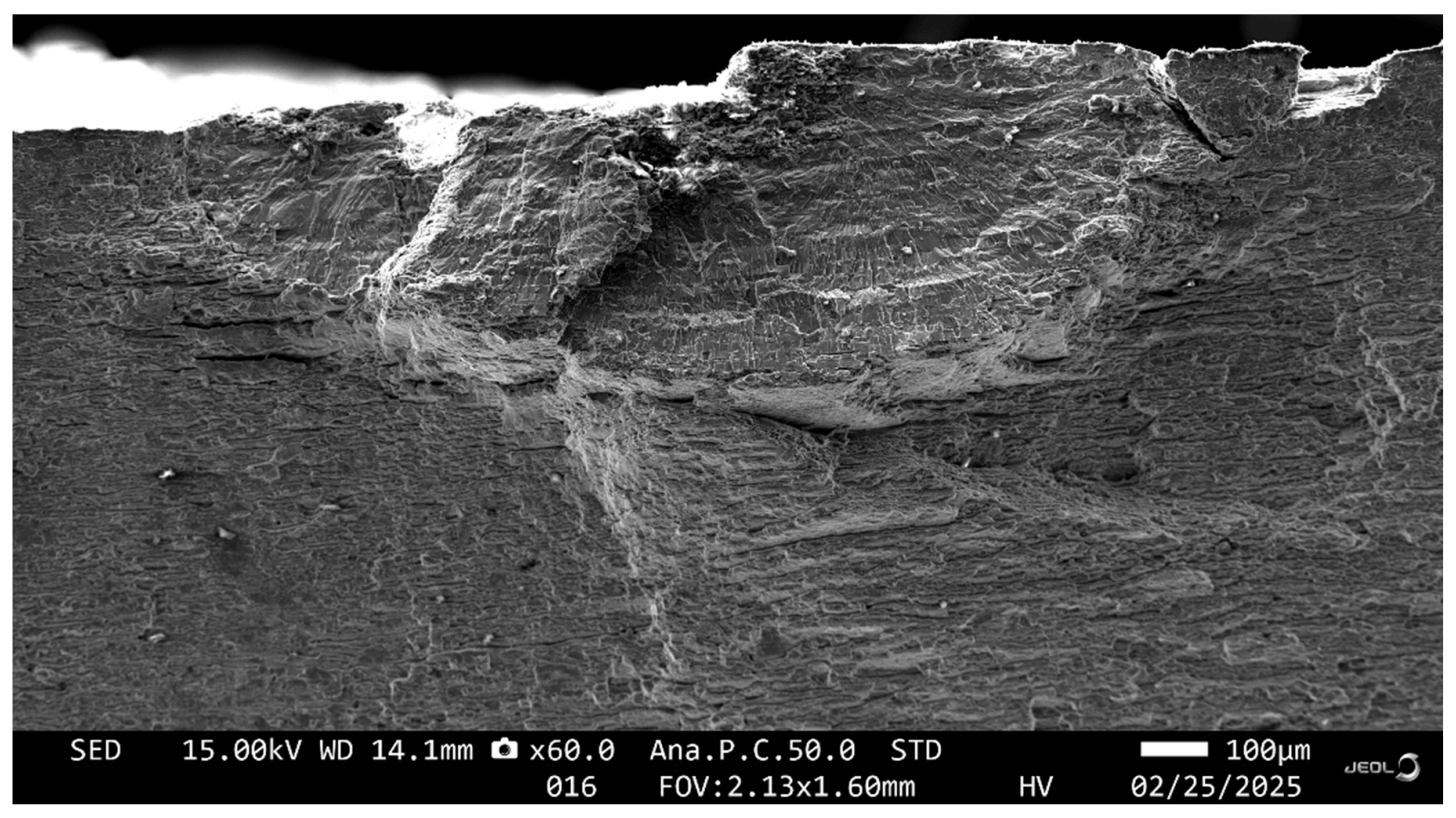
Local details of Crack 4.

SEM pictures of the nucleation sites associated with Cracks 1–4 are shown in Figure 22, Figure 23, Figure 24 and Figure 25. Although Crack 1 was not analysed, the SEM picture is included since it shows the presence of a number of intergranular cracks; see Figure 22. Whilst these intergranular cracks do not appear to be associated with the primary failure surface, they nevertheless further illustrate the propensity of AA7085-T7452 to exhibit intergranular cracking. In this context it should again be stressed that this failure mechanism has not previously been reported.

**Figure 26:**
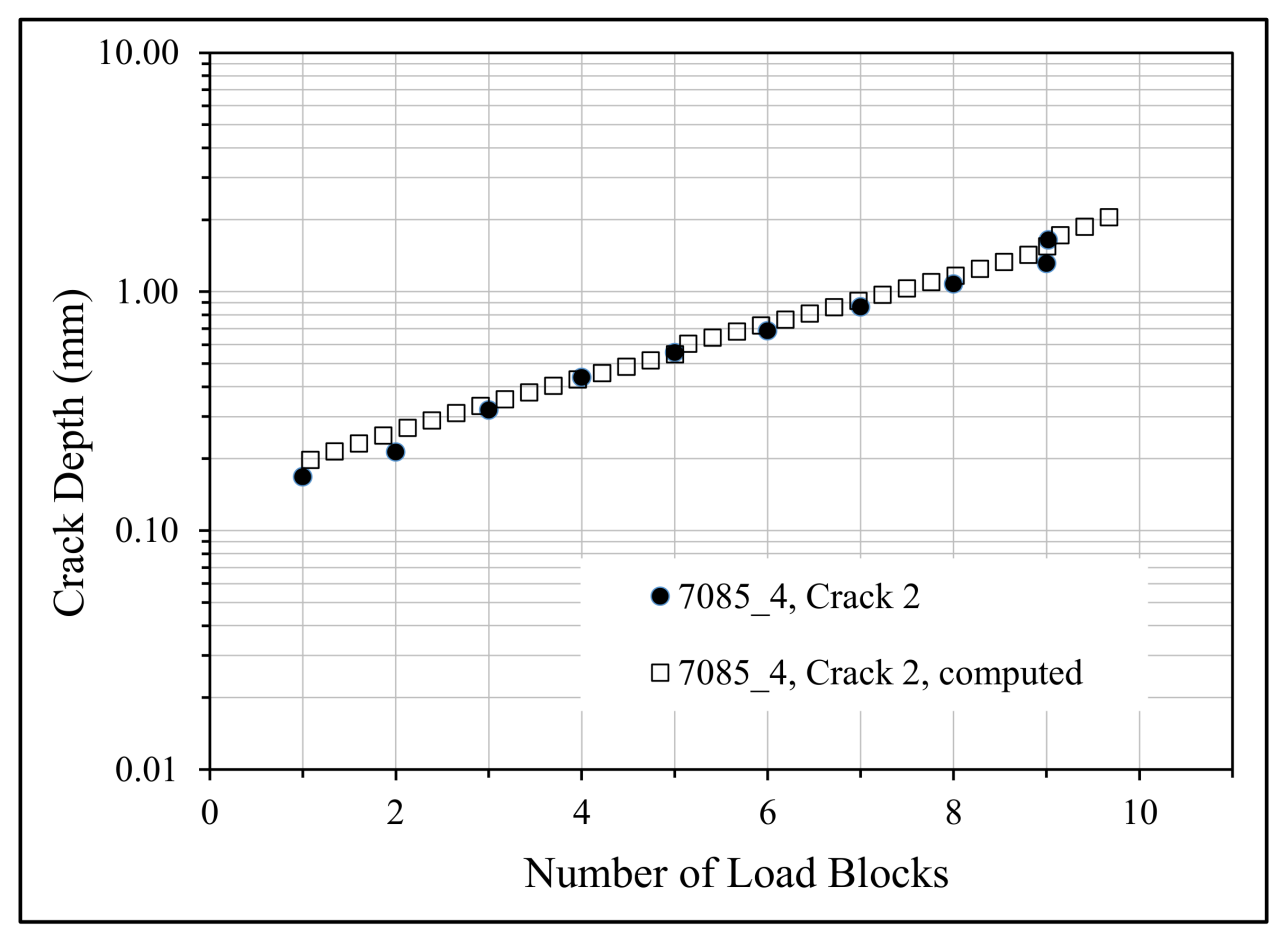
The measured and predicted curves associated with the Crack 2 in Specimen 7085_4.

**Figure 27:**
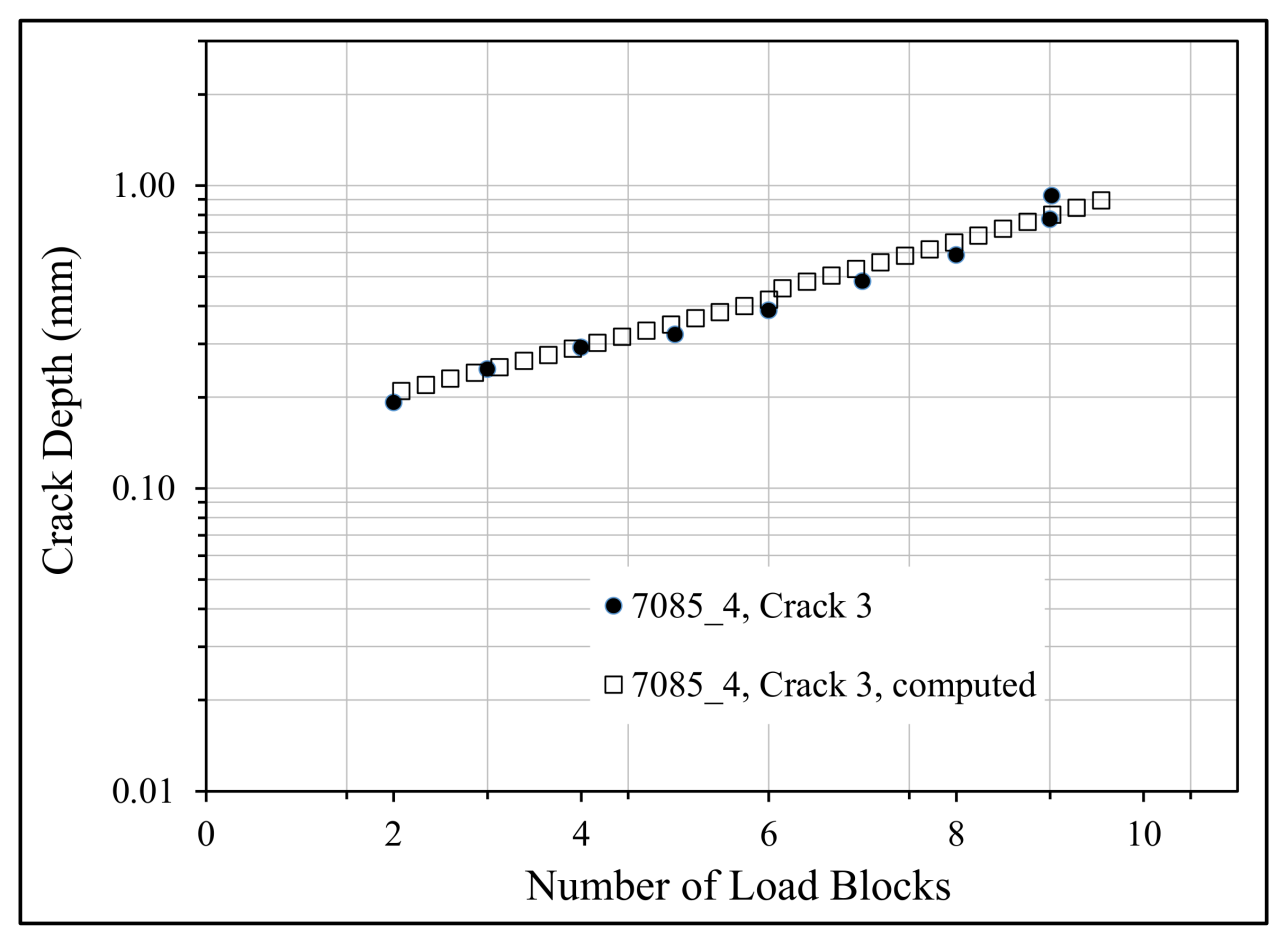
The measured and predicted curves associated with the Crack 3 in Specimen 7085_4.

**Figure 28:**
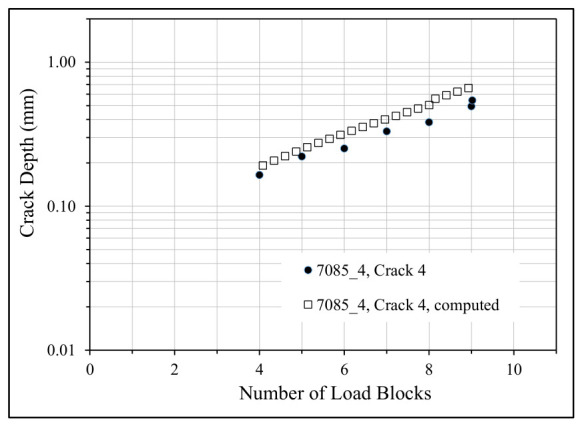
The measured and computed curves associated with the Crack 4 in Specimen 7085_4.

The measured crack growth histories, up to the transition from a classical Mode I crack to a layered-like failure surface, are shown in Figure 26, Figure 27 and Figure 28. At this point, Crack 2 had a depth of approximately 2.1 mm. This finding when taken in conjunction with Figure 19, Figure 20, Figure 21, Figure 22, Figure 23, Figure 24 and Figure 25 again suggests that failure was not a simple Mode I failure and hence failure was not due to the value of *K* exceeding its Mode I fracture toughness.

This observation, which will be discussed in more detail in Section 4, supports that reported in Section 3.2.1 and, as such, has implications with respect to the structural integrity and the airworthiness of aircraft structures that are fabricated from AA 7085-T7452.

### 3.3. Comparison of the Crack Growth Histories

**Figure 29:**
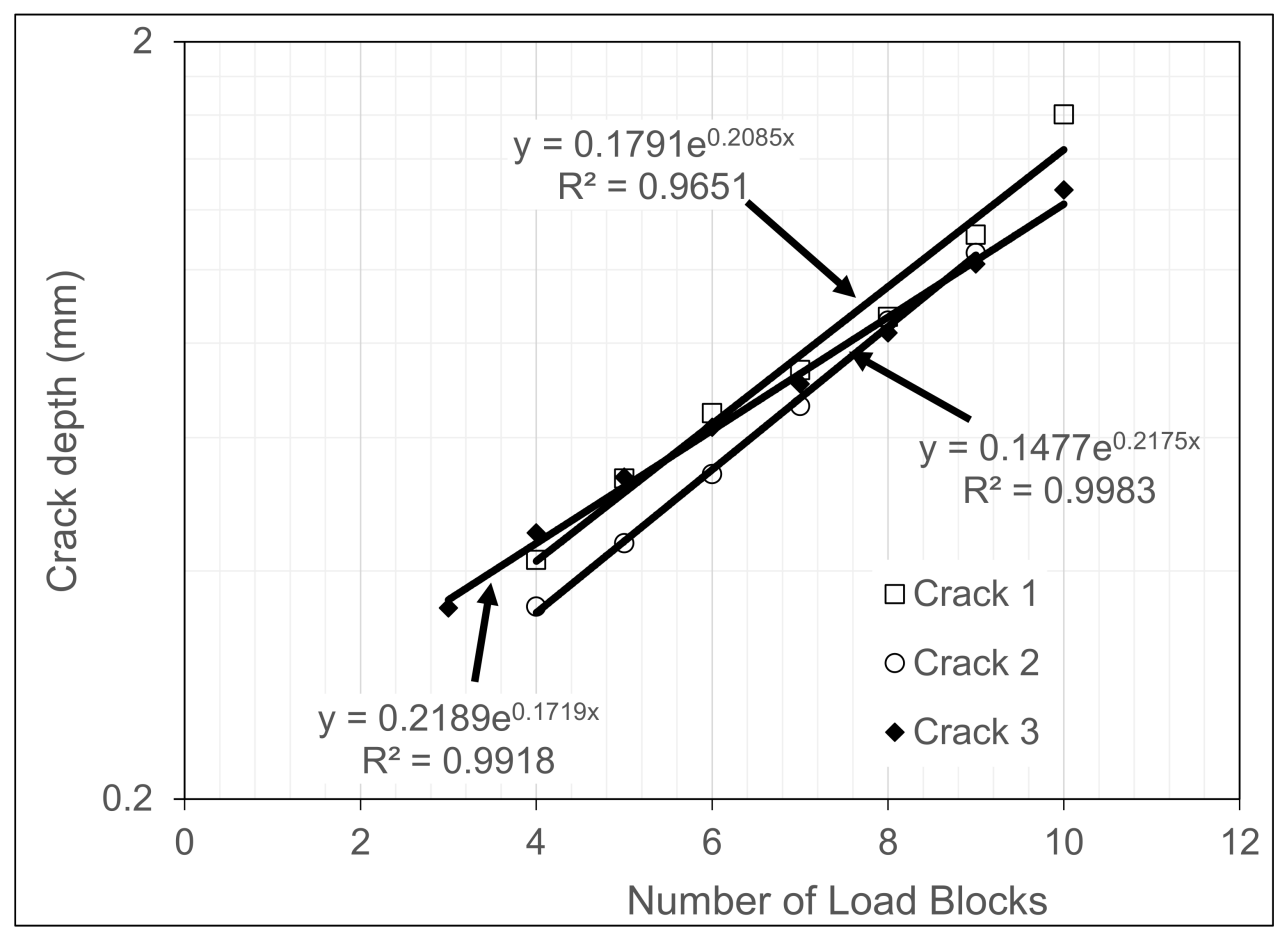
The crack growth histories associated with Specimen 7085_1. Note that data associated with the crack depth just prior to when the cracks changed planes was omitted.

**Figure 30:**
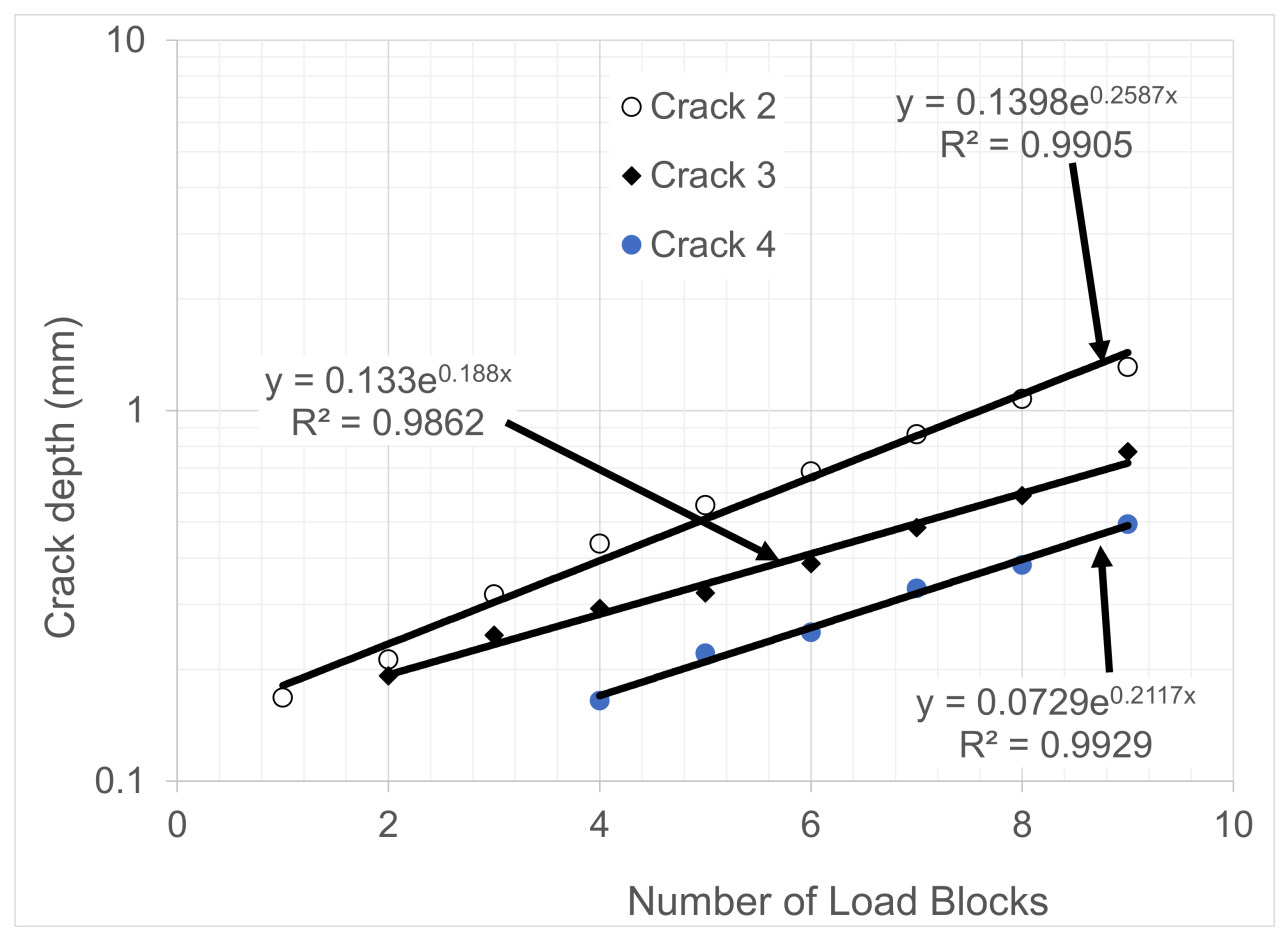
The crack growth histories associated with Specimen 7085_4. Note that data associated with the crack depth just prior to when the cracks changed planes was omitted.

The crack growth histories seen in Specimen 7085_1 are compared in Figure 29. The crack growth histories seen in Specimen 7085_4 are compared in Figure 30. (Here it should be noted that these plots are confined to data points associated with crack depths below the depth immediately prior to the point when the cracks changed planes. By this we mean in the region where the cracks behaved like a small 3D Mode I fatigue crack.)

Figure 29 reveals that the crack growth rates associated with Cracks 1 and 2 in Specimen 7085_1 are similar and that Crack 3 grows slower than Cracks 1 and 2. Figure 30 reveals that for Specimen 7085_4, the crack growth rate associated with Crack 2 is slightly faster than that associated with Crack 4 whilst Crack 3 grows slower than Cracks 2 and 4. This level of variability is to be expected in tests on the growth of small naturally occurring 3D cracks.

It is also important to note that in each case, the Mode I crack growth histories can, to a first approximation, be expressed as per [21,22,23,36,38,39,40,41,42,43,44,45,46,47,48,49,50,51,52,53,54,55,56,57,58,59] in the form

*a* = *a*_0_ e^(*FB*)^ (2)

where *a* is the crack depth, *B* is the number of load blocks, *F* is a constant, and *a*_0_ is the Equivalent Initial Damage Size (EIDS). (Here it should be recalled that in [36], the tests were on AA7050-T7451 specimens that were subjected to both pre-exposure and repeated intermittent fatigue and environmental exposure.)

Ignoring the slowest cracks in these tests, it can be seen that the exponents (*F*) of the (fastest) crack growth histories seen in these two tests lie in the range 0.21 to 0.26. In other words, considering the significant variability that is associated with the growth of small naturally occurring 3D cracks, and the fact that in both tests, the cracks transitioned at similar depths, the Mode I crack growth histories determined in these tests were reasonably consistent. These observations further reinforce the implications of these tests to the structural integrity assessment of aircraft structures that are fabricated from AA 7085-T7452.

It is also important to recall that in [57], Rudd et al. explained that the EIDS is a unique measure of the effect of the environment on a material’s economic life. Indeed, MIL-STD-1530D [60] mandates that any durability analysis should assume a minimum EIDS of 0.254 mm (0.01 inch). That said, the 2025 Lincoln Lecture at the US Aircraft Structural Integrity Program Conference ASIP25 [61] highlighted that, in the absence of environmental effects, the EIDS associated with naturally occurring cracks in aerospace aluminium alloys is generally much smaller than 0.254 mm. Figure 29 and Figure 30 reveal that the EIDS seen in the present tests were in the range 0.08 to 0.22 mm. Interestingly, the range of the EIDS seen in the present tests is comparable to the range in the EIDS seen in [36] for tests on 7050-T7451 that were subjected to both pre-exposure and repeated intermittent fatigue and environmental exposure. Rudd [57] would suggest that this observation, i.e., their EIDS, reflects the effect of the environment on the performance of these two materials. However, this clearly does not account for the effect that AA7085-T7452 has a somewhat unusual failure once *K* exceeds it interlayer fracture toughness, which we will define as *K_IL_*.

## 4. Computing the Crack Growth Histories in the AA 7085-T7452 Tests

### 4.1. The Computed Crack Growth History Associated with Specimen 7085_1

In this analysis, the initial size of Crack 1 was taken to be as per the first clearly measurable marker band, viz: a_0_ = 0.414 mm and c_0_ = 0.363 mm. Similarly, the initial size of Crack 2 was taken to be a_0_ = 0.359 mm and c_0_ = 0.496 mm. The initial size of Crack 3 was taken to be a_0_ = 0.358 mm and c_0_ = 0.413 mm. In all cases, “a” is the crack depth, as measured from the upper surface of the specimen, and c is the crack half-width.

The resultant predicted crack growth histories, which were predicted as outlined in Section 2, are shown in Figure 15, Figure 16 and Figure 17 where they are compared to the measured crack growth histories. These figures reveal that, up to the point where the crack transitions from a three-dimensional Mode I crack, the measured and predicted crack growth histories are in reasonable agreement.

That said, as can be seen in Figure 13 and Figure 14, once Cracks 2 and 3 reached depths of approximately 2.4 mm, the cracks turned to be at approximately ninety degrees to the initial Mode I plane. We will define the value of the stress intensity factor (*K*) at this depth to be *K_IL_*. The analysis revealed that, at this stage, the stress intensity factor at the deepest point was approximately 22 MPa √m. It is interesting to note that this value is significantly beneath the fracture toughness for this specimen, viz: 65 MPa √m, which was determined in [1] for this batch of AA7085-T7452. *This suggests that failure was not due to the value of K exceeding its Mode I fracture toughness.*

This finding is particularly important since the airworthiness certification of military and civil aircraft requires a knowledge of the critical crack length and hence a knowledge of the stress intensity factor at which the crack will fail. This latter value is referred to as the fracture toughness (*K_IC_*) of the material. The value *K_IC_* used to assess material failure, and hence the critical crack length, is based on tests on long crack specimens as described in the main body of the fatigue test standard ASTM E399 [62]. However, this standard is associated with failures where the direction of failure is in the same direction as the initial crack (i.e., a self-similar failure). Since the current specimens did not fail in this fashion, and since the value of the stress intensity factor at failure was found to be significantly below the value of *K_IC_* reported in the literature, this means that the hypothesis that the value of *K_IC_* as determined by standard ASTM E399 tests can be used to comply with the airworthiness certification requirement cannot be assumed to be true. What this means is that whilst there may be instances where, for AA7085-T7452 components, this hypothesis may hold, the current test results reveal that it cannot be assumed to be true. As a result, further research is needed into when the traditional design hypothesis holds and when it is not applicable. This discovery has not previously been documented.

#### A Rough Check on the Value of *K_IL_*

Given the spacing between the cracks, the depth of the cracks relative to the depth of the test specimen, and the results presented in [46] for interacting 3D cracks, a first estimate of the stress intensity factor *K_IL_* can be obtained by ignoring both finite geometry and crack interaction effects. This yields an approximate value of

*K_IL_* ≈ 400 × 2 × √(3.1415 × 2.4/1000)/3.1415 = 22.5 MPa √m.

This approximate estimate is consistent with the values obtained in the analysis.

### 4.2. The Computed Crack Growth History Associated with Specimen 7085_4

The CAD model for this specimen is shown in Figure 7 and the mesh used to compute the stress intensity factors is shown in Figure 9. The crack growth analysis again used the approach described in Section 2. The initial starting size of the cracks, which we have defined as a_i_ and c_i_, in the analysis of Specimen 7085_4 were again taken to be as per the first clearly measurable marker band, viz:

(i) For crack 2: a_i_ = 0.168 mm and c_i_ = 0.367 mm;
(ii) For crack 3: a_i_ = 0.192 mm and c_i_ = 0.204 mm;
(iii) For crack 4: a_i_ = 0.165 mm and c_i_ = 0.328 mm;

The resultant predicted crack growth histories are shown in Figure 26, Figure 27 and Figure 28 where they are compared to the measured crack growth histories. These figures reveal that, up to the point where the crack transitions from a three-dimensional Mode I crack, the measured and predicted crack growth histories are in reasonable agreement.

In the case of Specimen 7085_4, the crack depth at which the crack turned from the plane of the initial fatigue crack was approximately 2 mm. At this depth, the computed value of the stress intensity factor (*K*), which we have termed *K_IL_*, was approximately 20 MPa √m. This value is also significantly beneath the fracture toughness for this specimen, viz: 65 MPa √m, which was determined in [1] for identical specimens that were built from this batch of AA7085-T7452.

As such, these tests again suggest the possibility that, at least for these pre-corroded specimens, there is what we will term a minimum “interlayer fracture toughness” *K_IL_* of approximately 20 MPa √m. This minimum value is significantly lower than the Mode I fracture toughness. It would appear that when the stress intensity factor reached *K_IL_*, the crack turned to be at a (near) 90° angle to the initial Mode I crack.

This finding has implications with respect to aircraft structures that are fabricated from AA 7085-T7452.

## 5. Thoughts on the Unusual Failure

Exposure in the B117-19 environmental chamber resulted in the creation of pits with depths that were of the order of 0.4 mm deep. Similar pit depths were also found in the author’s prior test programme [1] as well as in prior tests on corroded AA 7050-T7451 specimens [36]. These pit depths were slightly greater than those reported in [38] after twelve months of outdoor exposure of 7085-T7452 specimens to a hot–humid marine environment on Hainan Island, China [38]. These pit depths are also consistent with those reported in AA 7050-T7451 airframes in RAAF F/A-18 A/B aircraft [26,32,33]. As such, it is believed that the results of this test programme may be particularly relevant to operational aircraft.

It is suggested that the unusual crack path seen in these tests is not driven by a single factor but by a number of possible and compounding factors.

### 5.1. Weak Interlayer Strength

Examining Figure 13, Figure 14, Figure 19 and Figure 20, it is clear that AA7085-T7452 has a weak interlayer strength. Despite the efforts made prior to fatigue testing to minimise the effect of the prior exposure on the subsequent fatigue tests, the role of hydrogen embrittlement cannot be ruled out. That said, it should be noted that in the test programme discussed in [36] where not only were AA7050-T7451 specimens pre-exposed to an aggressive environment but the fatigue tests were regularly stopped and the specimens re-exposed for a week, there was no evidence of the effect of the environment on crack growth. In other words, there was no evidence of pre-exposure causing hydrogen embrittlement.

### 5.2. The Crack Path: T-Stress and Crack Path Stability

The paper by Sih [63] and the subsequent paper by Eftis et al. [64] revealed that the stress field ahead of a crack tip is a function of both the stress intensity factor (*K*) and a non-singular term that is known as the T-stress. Indeed, the experimental results presented in Eftis et al. [64] revealed that the T-stress can make a significant difference to the direction of crack growth. Furthermore, according to Cotterell and Rice [65], the sign and magnitude of the T-stress can dictate whether a crack path remains straight (flat) or results in an unstable crack path.

Consequently, it is hypothesised that this unusual interlayer failure may also be due to a complex interaction between both the stress intensity factor and the T-stress and the weak interlayer toughness of the material. However, further studies are needed to evaluate this hypothesis.

### 5.3. The Potential Role of Hydrogen-Assisted Cracking

Chen et al. [66] noted that the electrochemical conditions at the base of pits that are created in this fashion, i.e., by pre-exposure, can result in enhanced hydrogen embrittlement at the base of the pit. (Interesting discussions on the susceptibility of 7085 to hydrogen embrittlement can be found in Song et al. [67] and in Yang et al. [68]. That said, the paper by Košová Altnerová et al. [69] is also particularly interesting in that it discusses hydrogen embrittlement in a much wider range of aluminium alloys.) Chen et al. [66] also explained that subsequent fatigue testing and the associated high stress gradient ahead of the pitting, and subsequently ahead of the crack tip, can also result in highly mobile atomic hydrogen diffusing into the metal lattice and being preferentially trapped at the elongated grain boundaries. This high hydrogen concentration can degrade the cohesive atomic bonds—a process known as Hydrogen-Enhanced Decohesion (HEDE).

That said, it is unclear if hydrogen embrittlement played a significant role in the failures seen in the current test programme. Indeed, a more focused study is needed to evaluate this aspect. However, until this is done, the role of hydrogen embrittlement cannot be ruled out.

## 6. Conclusions

The paper is the first to highlight what appears to be a unique and unusual failure mechanism that is associated with cracks that nucleate from corrosion pits in AA 7085-T7452 aluminium alloy specimens. In each test, a number of cracks nucleated at corrosion pits. These cracks first grew, as would be expected, as a small Mode I fatigue crack at ninety degrees to the surface and perpendicular to the applied load. However, after reaching a depth of approximately two mm, the cracks turned to be at a near 90° angle to their initial Mode I plane. The values of the stress intensity factors (*K_IL_*) at which this phenomenon occurred were reasonably consistent. That said, in each case, the values were significantly less than the fracture toughness for this material. In other words, final failure would appear to be associated with a weak interlayer toughness (*K_IL_*).

This crack growth process is not a classical Mode I mechanism and failure occurred when *K* exceeded *K_IL_*. It is conjectured that this failure may be a function of both the stress intensity factor and the T-stress. This observation would appear to counter the common hypothesis, which is embedded in the damage tolerance assessment of AA7085-T7452 parts, that failure occurs when the stress intensity factor (*K*) exceeds its fracture toughness (*K_IC_*).

As such, the international community is challenged to repeat these tests. If this unusual failure mechanism is confirmed, then there are also implications with respect to the sustainment of civil and military aircraft built using AA7085-T7452.

Despite the unusual failures, it was found that, up to the point where this phenomenon occurred, not only was the growth of the fastest growing (lead) cracks in these two tests consistent, but the crack growth versus cycles histories could be reasonably accurately predicted using the small crack growth equation developed by the authors in a previous study on cracks that nucleated from corrosion damage in tests that were performed on AA 7085-T7452 specimens with a fastener hole. As in the authors’ previous study into cracking in AA7085-T7452, the fatigue threshold term in the current analyses was set to be approximately 0.1 MPa √m. This value is significantly lower than the values of *K_Iscc_* reported for AA7085-T7452.

The materials science community is challenged to repeat these tests and to explain the underlying science.

## Figures and Tables

**Table 1:** Values of the yield stress (σ_y_), ultimate strength (σ_ult_), and strain to failure of die-forged AA7085-T7452.

σ_y_ (MPa)	σ_ult_ (MPa)	Strain to Failure (mm/mm)
448–462	496–503	0.07–0.10

## Data Availability

The original contributions presented in the study are included in the article. Further inquiries can be directed to the corresponding author.
